# Transcription factor NFYA3_0 promotes MTA-mediated m^6^A modification of PVY genomic RNA to confer antiviral resistance in *Nicotiana benthamiana*

**DOI:** 10.1016/j.xplc.2025.101584

**Published:** 2025-10-31

**Authors:** Jiao Li, Jianli Luo, Hongfu He, Fenyan Wang, Huan Wu, Chunni Zhao, Runjiang Song, Baoan Song

**Affiliations:** State Key Laboratory of Green Pesticide, Guizhou University, Guiyang 550025, P.R. China

**Keywords:** antiviral immunity, *N*^6^-methyladenosine, methyltransferase, nuclear transcription factor, potato virus Y

## Abstract

*N*^6^-methyladenosine (m^6^A), a reversible epigenetic modification, is ubiquitously present across diverse RNA species, including viral RNA. This modification plays a pivotal role in orchestrating RNA metabolism. Nevertheless, the mechanisms by which plants utilize m^6^A modifications to fine-tune antiviral immunity remain largely unknown. In this study, we systematically examined the dynamic changes and biological significance of m^6^A modifications throughout potato virus Y (PVY) infection in host plants. Methylated RNA immunoprecipitation sequencing reveals a conserved m^6^A distribution pattern in *Nicotiana benthamiana*, predominantly enriched in 3′ untranslated regions of transcripts. The nuclear transcription factor NFYA3_0 exhibits robust hypermethylation accompanied by transcriptional upregulation during PVY infection. *NFYA3_0* knockdown promotes PVY accumulation and reduces global m^6^A modification levels in the host. NFYA3_0 positively regulates transcription of the m^6^A methyltransferase gene *NbMTA*, whose loss of function similarly compromises viral resistance while diminishing m^6^A abundance. Moreover, NbMTA anchors and methylates the PVY coat protein-coding region, thereby facilitating viral RNA degradation and effectively restricting infection. These findings indicate that the core antiviral mechanism of the nuclear transcription factor NFYA3_0 enables targeted degradation of viral RNA by activating methyltransferase NbMTA-mediated m^6^A epitranscriptome regulation. This work provides a new strategy for developing green virus-resistant crop lines based on epigenetic editing.

## Teaser

This study shows that the transcription factor NFYA3_0 is upregulated during PVY infection and activates the m^6^A methyltransferase NbMTA, which then deposits m^6^A modifications directly onto the viral RNA, targeting it for degradation and restricting viral replication. These findings establish a novel NFYA3_0–NbMTA–m^6^A pathway for antiviral defense.

## Introduction

*N*^6^-methyladenosine (m^6^A), the most prevalent internal RNA modification in eukaryotic systems, constitutes a dynamically reversible epigenetic marker that regulates RNA metabolism across diverse biological contexts (He and He, 2021; Jiang et al., 2021). The methyltransferase complex in plants operates through a conserved catalytic core comprising the methyltransferase-like proteins MTA (a METTL3 homolog) and MTB (a METTL14 homolog), which require auxiliary components, including FKBP12-interacting protein 37, VIRILIZER, and HAKAI/CBLL1, to form functional m^6^A deposition machinery (Růžička et al., 2017; Bhat et al., 2020). This sophisticated enzymatic apparatus coordinates with RNA-binding partners to precisely recognize substrate transcripts and mediate their methylation to determine their fates; opposing demethylase enzymes (“erasers”) actively remove these modifications, establishing a dynamic regulatory circuit (Yue et al., 2019; Hou et al., 2022). m^6^A modification functions as a flexible regulatory system that links environmental signals with developmental processes, playing a central role in how plants adapt to both biotic and abiotic stressors. It thus serves as a critical focus for understanding epigenetic regulation of stress-responsive gene expression.

Advances in m^6^A detection technology have shifted the focus from broad epitranscriptomic profiling to detailed functional analysis of specific modification sites. Increasing evidence indicates that m^6^A modification influences many biological processes in mammals, such as innate immune responses (Zheng et al., 2017), sex determination (Lence et al., 2016), meiosis (Xu et al., 2017), cancer (Sun et al., 2019), and viral life cycles (Imam et al., 2018). Additionally, plant researchers have shown that m^6^A exhibits both shared and unique functions in regulating growth, development, and evolutionary adaptation (Zhong et al., 2008; Bodi et al., 2012; Hu et al., 2024). Notably, the *Arabidopsis* m^6^A eraser AtALKBH9B assists alfalfa mosaic virus spread by directly interacting with the virus’s coat proteins (Martínez-Pérez et al., 2017). In rice, m^6^A patterns shift during infection with rice stripe virus and rice black streaked dwarf virus, suggesting that these viruses can hijack the plant epitranscriptomic system (Zhang et al., 2021). Genome-wide association studies identified TaMTB as a proviral factor that enhances wheat yellow leaf virus susceptibility in wheat (Zhang et al., 2022; Ge et al., 2025). Conversely, the plant m^6^A reader protein ECT2 recognizes m^6^A-modified viral RNA and recruits the nonsense-mediated decay factors UPF3 and SMG7, leading to viral RNA breakdown and reduced infection. This reveals a plant antiviral mechanism in which m^6^A marks viral RNA for degradation through the nonsense-mediated decay (NMD) pathway (He et al., 2024). Although m^6^A modification has emerged as a key molecular regulator balancing plant–virus interactions, the mechanisms by which it aids plant responses to viral stress have not been fully elucidated.

Nuclear transcription factors (NF-Y), a class of transcription factors predominantly found in higher eukaryotes, are also known as CCAAT-binding factors and heme activator proteins due to their specific binding to the CCAAT box (Dolfini et al., 2011; Laloum et al., 2013; Zhao et al., 2017). NF-Ys function as heterotrimeric complexes composed of three subunits (NF-YA, NF-YB, and NF-YC) (Mantovani, 1999; Kahle et al., 2005). Each subunit can interact with diverse transcription factors; together, they play key roles in regulating plant growth and development (Kumimoto et al., 2010). Emerging evidence suggests that NF-Ys also contribute to plant immune regulation. For example, in rice, NF-YA2 (encoded by *OsHAP2E*) enhances resistance to blast disease via *miR169a*-mediated regulation (Alam et al., 2014). In tomato, NF-Ys influence flavonoid biosynthesis by modulating chromatin structure through Histone H3 lysine 27 trimethylation remodeling (Wang et al., 2020). Crucially, NF-Ys have been shown to suppress antiviral defense in rice by inhibiting the jasmonate signaling pathway through interactions with the key transcription factors OsMYC2/3 (Tan et al., 2022). Whereas the roles of NF-Ys in defense against fungal and bacterial pathogens are well established, their potential involvement in m^6^A-mediated antiviral pathways remains largely unexplored.

Potato virus Y (PVY), a member of the *Potyvirus* genus, is one of the most economically devastating pathogens affecting global potato production (Quenouille et al., 2013; Wang et al., 2025). In this study, we identify a previously unrecognized NFYA3_0–NbMTA regulatory axis governing epitranscriptomic modifications in plant antiviral defense. The m^6^A deposition patterns in *Nicotiana benthamiana* (*N. benthamiana)* are evolutionarily conserved and predominantly enriched within 3′ untranslated regions (UTRs) of transcripts. Upon PVY infection, the *NFYA3_0* gene exhibits infection-responsive hypermethylation and increased transcriptional activity. Genetic knockdown of *NFYA3_0* potentiates viral accumulation and reduces global m^6^A modification levels in host plants, establishing a functional association between NFYA3_0 activity and epitranscriptomic regulation. Notably, NFYA3_0 acts as a transcriptional activator of the m^6^A methyltransferase gene *NbMTA*. Loss of *NbMTA* function mirrors the effects of *NFYA3_0* silencing, resulting in increased viral susceptibility and decreased m^6^A abundance. Mechanistic analyses indicate that the NbMTA protein selectively targets and methylates the PVY capsid protein (CP) coding region, thereby promoting site-specific viral RNA destabilization via m^6^A-mediated decay mechanisms. These findings provide a mechanistic instance of host plants leveraging the NFYA3_0–NbMTA regulatory axis to counteract viral pathogenesis and suppress infection progression through epitranscriptome-driven antiviral reprogramming.

## Results

### PVY infection induces dynamic m^6^A methylation changes in *N*. *benthamiana*

**Figure 1:**
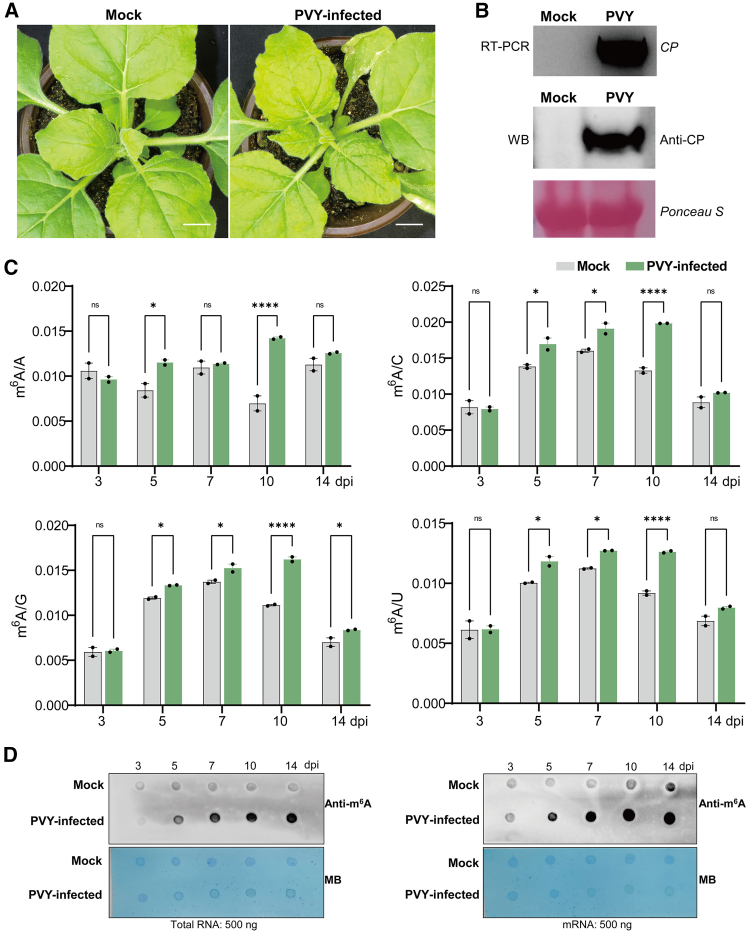
PVY infection induces dynamic changes in m^6^A methylation in *N*. *benthamiana*. **(A)** Symptoms of PVY infection in *N*. *benthamiana*. Scale bar: 3 cm. **(B)** RT-PCR and western blot analysis of PVY *CP* expression at 10 dpi. **(C)** LC–MS/MS detection of dynamic changes in m^6^A levels in mRNA during PVY infection in *N*. *benthamiana*. Asterisks indicate significant differences between mock- and PVY-infected plants based on two-way ANOVA (*∗∗*P < 0.01; *∗∗∗P* < 0.001; not significant [ns], *P* > 0.05). Comparison of overall m^6^A levels at different time points: m^6^A/A (3 dpi, *P* = 0.7813; 5 dpi, *P* = 0.0154; 7 dpi, *P* = 0.9921; 10 dpi, *P* < 0.0001; 14 dpi, *P* = 0.5293), m^6^A/C (3 dpi, *P* = 0.9994; 5 dpi, *P* = 0.0106; 7 dpi, *P* = 0.0117; 10 dpi, *P* < 0.0001; 14 dpi, *P* = 0.4436), m^6^A/G (3 dpi, *P* = 0.9990; 5 dpi, *P* = 0.0271; 7 dpi, *P* = 0.0178; 10 dpi, *P* < 0.0001; 14 dpi, *P* = 0.0359), m^6^A/U (3 dpi, *P* > 0.9999; 5 dpi, *P* = 0.0117; 7 dpi, *P* = 0.0353; 10 dpi, *P* < 0.0001; 14 dpi, *P* = 0.1459). Error bars represent standard deviation (SD; *n* = 2). **(D)** Dynamic changes in m^6^A levels in total RNA and mRNA during PVY infection, analyzed using an m^6^A dot blot assay, with 0.1% methylene blue staining for nucleic acid quantification.

We used PVY-infected *N*. *benthamiana* as a model host to measure m^6^A level changes and investigate the dynamics of m^6^A modification during PVY infection. In contrast to mock-treated plants, the top leaves exhibited evident wrinkling and mottling symptoms by 14 days post inoculation (dpi) (Figure 1A). Successful infection of *N*. *benthamiana* was confirmed using reverse transcription (RT)–polymerase chain reaction (PCR) detection of PVY RNA and immunoblot analysis of CP accumulation (Figure 1B). We performed liquid chromatography–tandem mass spectrometry (LC–MS/MS) analysis (Figure 1C and Supplemental Figure 1) and m^6^A dot blot assays (Figure 1D) on total RNA and mRNA isolated from systemic leaf tissues of PVY-infected plants at 3, 5, 7, 10, and 14 dpi to characterize the temporal dynamics of m^6^A modification during viral infection. Quantitative LC–MS/MS results revealed that all measured m^6^A ratios (A/C/G/U) in both RNA fractions were significantly elevated (*P* < 0.05) during the early to middle stages of infection (5–10 dpi) compared with those in mock-treated plants. The modification levels exhibited progressive accumulation, peaking at 10 dpi with a 1.37- to 2.04-fold increase relative to mock-treated plants, followed by a decline at 14 dpi. Notably, m^6^A dot blot analysis consistently demonstrated a parallel dynamic increase in global m^6^A levels in both total RNA and mRNA upon infection (Figure 1D), further supporting the LC–MS/MS findings. Taken together, these orthogonal datasets from two independent methodologies indicated that m^6^A modification participates in active regulatory mechanisms during PVY–host interactions.

### m^6^A methylation dynamics and transcriptomic response in *N*. *benthamiana* upon PVY infection

To obtain m^6^A-modified epigenetic transcriptome profiling of PVY-infected *N*. *benthamiana*, the upper freshly emerged leaves of 36 biological samples (18 mock- and 18 PVY-inoculated plants) were collected for the construction of a methylated RNA immunoprecipitation sequencing (MeRIP-seq) dataset with two independent biological replicates. After stringent quality control procedures, including adapter trimming, low-quality read removal, and rRNA depletion, each replicate yielded approximately 53–59 million high-quality clean reads, with 89.91%–94.42% of reads uniquely mapped to the *N*. *benthamiana* Nbe1.01 reference genome (Supplemental Figure 2). We implemented a comprehensive validation strategy to ensure data reliability: (1) inter-sample Pearson correlation analysis demonstrated high reproducibility (*R* ≥ 0.94; Supplemental Figure 3), and (2) m^6^A immunoprecipitation (IP)–quantitative PCR (qPCR) validation of randomly selected candidate genes confirmed strong concordance between the predicted methylation regions and experimental results (Supplemental Figures 4A–4C). These systematic validations robustly demonstrated the high quality of the MeRIP-seq dataset, establishing its suitability for investigating PVY infection-induced epitranscriptomic regulatory mechanisms.

**Figure 2:**
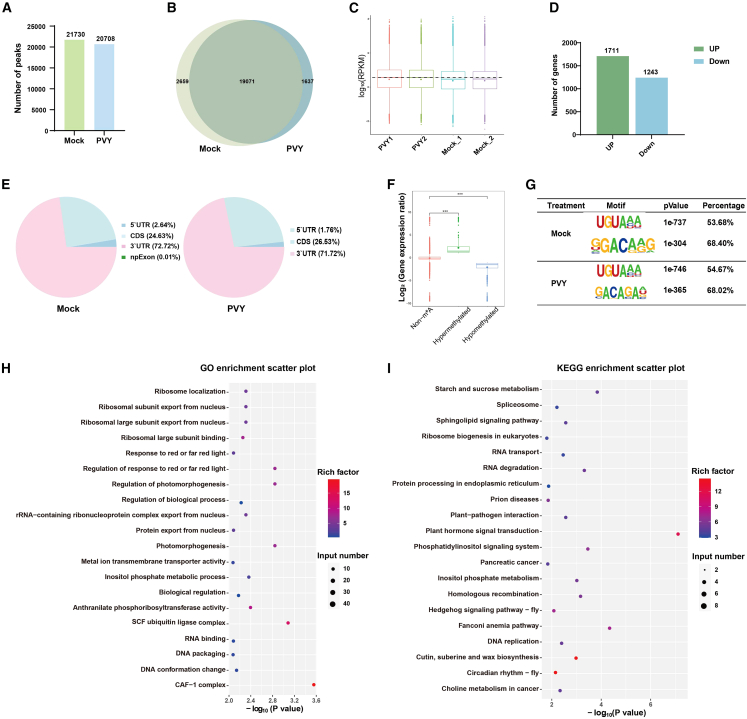
Epitranscriptome analysis of m^6^A modification in *N. benthamiana* after PVY infection. **(A)** Number of m^6^A peaks in mock- and PVY-infected *N*. *benthamiana*. **(B)** Venn diagram showing m^6^A peaks identified in mock- and PVY-infected plants. **(C and D)** Overall gene expression levels after PVY infection. **(E)** Pie chart showing distribution of m^6^A peaks across genomic elements. **(F)** Boxplot comparison of m^6^A-modified and non–m^6^A-modified transcript expression under healthy and PVY-infected conditions (*∗∗∗P* < 0.001, Wilcoxon test). **(G)** m^6^A motif analysis plot generated by extracting peak-associated sequences with HOMER, scanning for shared motifs, and visualizing conserved m^6^A-modified sequences in *N*. *benthamiana* under healthy and PVY-infected conditions. **(H)** GO analysis scatterplot of differentially methylated genes. **(I)** KEGG pathway analysis scatterplot of differentially methylated genes.

Global profiling of m^6^A methylation identified distinct landscapes in *N*. *benthamiana* under PVY challenge, delineating the epigenetic remodeling induced by viral infection. Mock-treated plants exhibited 21,730 m^6^A peaks, whereas PVY-infected plants showed 20,708 peaks; 19,071 peaks were shared between conditions. Notably, 2,659 mock-treatment-specific and 1,637 PVY-specific peaks were detected (Figure 2A and 2B). Transcriptomic analysis further revealed significant transcriptional activation in PVY-infected plants, characterized by 2954 differentially expressed genes (1,711 upregulated, 1,243 downregulated; log_2_(fold change) ≥ 1 or ≤ −1, *P* < 0.05) (Figure 2C and 2D). Spatial mapping demonstrated that m^6^A peaks were preferentially localized to the 3′ UTR and coding sequences in both groups, indicating no infection-induced change in positional specificity (Figure 2E and Supplemental Figure 5). Strikingly, the combined analysis of RNA sequencing (RNA-seq) and m^6^A-seq showed that hypermethylated genes exhibited substantially elevated expression compared with hypomethylated counterparts, underscoring a robust positive correlation between m^6^A modification levels and transcriptional activity (Figure 2F).

Functional enrichment analyses were performed to clarify the biological and evolutionary importance of these modifications. Gene Ontology (GO) terms highlighted roles in biological process regulation, RNA binding, and DNA packaging (Figure 2H), whereas Kyoto Encyclopedia of Genes and Genomes (KEGG) pathway analysis implied that RNA degradation, plant–pathogen interaction, and hormone signal transduction served as key mechanisms modulated by PVY infection (Figure 2I). Genomic mapping revealed a broad distribution of m^6^A peaks across the genomic scaffolds, such that more than 90% of transcripts harbored a single peak (Supplemental Figures 6A and 6B). Notably, hypergeometric motif analysis showed that the plant m^6^A sequence motifs UGUAYA (Y = A/C/U) and RRACH (R = A/G/C; H = A/G) were highly enriched, sharing homology with motifs identified in *Arabidopsis* (UGUAY and RRACH) and maize (UGUAMM) (Figure 2G) (Wei et al., 2018; Luo et al., 2019; Cai et al., 2024). These findings collectively indicate that PVY infection reconfigures the host transcriptional program via m^6^A-mediated regulation while conserving core methylation positional rules. The conserved m^6^A motifs further underscore the evolutionary conservation of m^6^A machinery in plant antiviral responses, potentially facilitating viral adaptation through regulation of RNA stability and hormonal signaling crosstalk.

### The nuclear transcription factor NFYA3_0 is involved in the m^6^A modification pathway and influences viral infection

**Figure 3:**
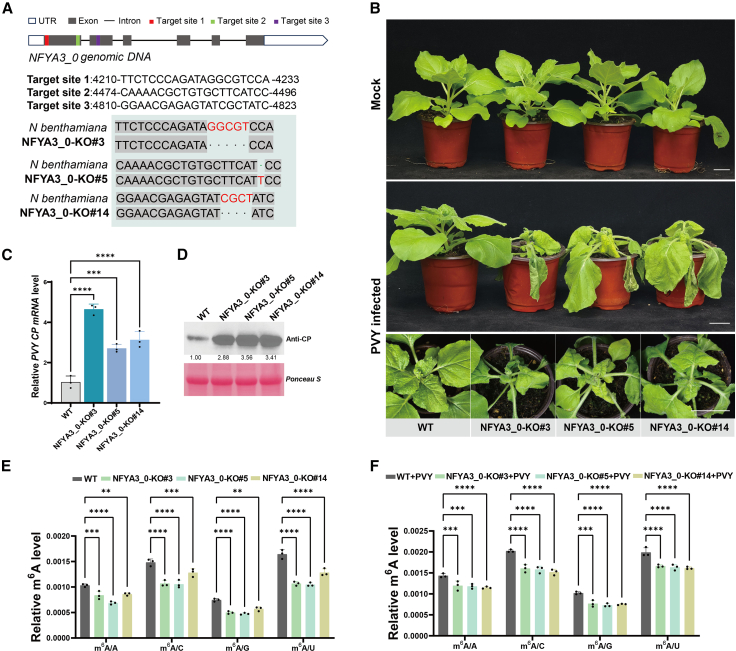
NFYA3_0 inhibits viral invasion through the m^6^A modification pathway. **(A)** Schematic illustration of the three target sites designed for *NFYA3_0* KO and validation of the corresponding *NFYA3_0* KO lines using PCR-based sequencing. **(B)** Diagram showing mock or PVY infection after *NFYA3_0* KO. Scale bar: 5 cm. **(C)** RT-qPCR analysis of relative viral expression levels in **(B)**. Asterisks indicate significant differences based on one-way ANOVA (∗∗*P* < 0.01; ∗∗∗*P* < 0.001; ∗∗∗∗*P* < 0.0001). Cumulative viral loads in different *NFYA3_0* KO strains were compared with those in the WT (*P* < 0.001, one-way ANOVA). Error bars represent SD (*n* = 3). **(D)** Western blot analysis of relative viral protein expression in **(B)**. **(E)** and **(F)** LC–MS/MS detection of overall m^6^A levels in mRNA from healthy and PVY-infected *N*. *benthamiana*, including WT and various *NFYA3_0* KO lines. Asterisks indicate significant differences based on two-way ANOVA (∗∗ *P* < 0.01; ∗∗∗*P* < 0.001; ∗∗∗∗*P* < 0.0001). Error bars represent SD (*n* = 3).

MeRIP-seq profiling identified six genes exhibiting concomitant hypermethylation and upregulation of mRNA expression, including *NFYA3_0*, which encodes an NF-Y subunit. As a phylogenetically conserved CCAAT box binder in higher eukaryotes, NF-Y complexes regulate pleiotropic processes spanning developmental programming and stress-responsive transcriptomes (Kumimoto et al., 2010). We established a tobacco rattle virus–mediated gene silencing system (TRV-VIGS) targeting *N*. *benthamiana NFYA3_0* and generated CRISPR-Cas9–mediated *NFYA3_0* knockout (KO) lines to investigate the role of *NFYA3_0* in m^6^A mRNA modification and viral infection. Successful gene silencing and KO were validated (Supplemental Figures 7A and 7B and Figure 3A). Pathophenotypic analysis showed exacerbated leaf wrinkling and bending in *NFYA3_0*-deficient plants at 7 dpi with PVY relative to the controls (Figure 3B and Supplemental Figure 7D). Consistent with these results, RT-qPCR and western blot analyses detected elevated PVY RNA accumulation and increased viral coat protein levels in *NFYA3_0*-compromised plants (Figure 3C and 3D and Supplemental Figures 7E and 7F), indicating impaired antiviral immunity. Global m^6^A level analysis via LC–MS/MS showed that *NFYA3_0*-knockdown (*TRV-NFYA3_0*) plants exhibited significantly reduced mRNA m^6^A levels under healthy conditions compared with the control (TRV-GUS) (Supplemental Figure 7C). Moreover, comprehensive analysis of independent *NFYA3_0* KO lines demonstrated consistent and significant decreases in m^6^A modification levels in both total RNA and mRNA under healthy conditions, as well as under PVY infection, relative to wild-type (WT) controls (Figures 3E and 3F and Supplemental Figure 8). These findings demonstrated that *NFYA3_0* is essential for maintaining m^6^A homeostasis in plants under both normal and stress conditions. Collectively, these results confirm that *NFYA3_0* functions as a key m^6^A regulator and influences the course of viral infection.

### NFYA3_0 activates *NbMTA* transcription

**Figure 4:**
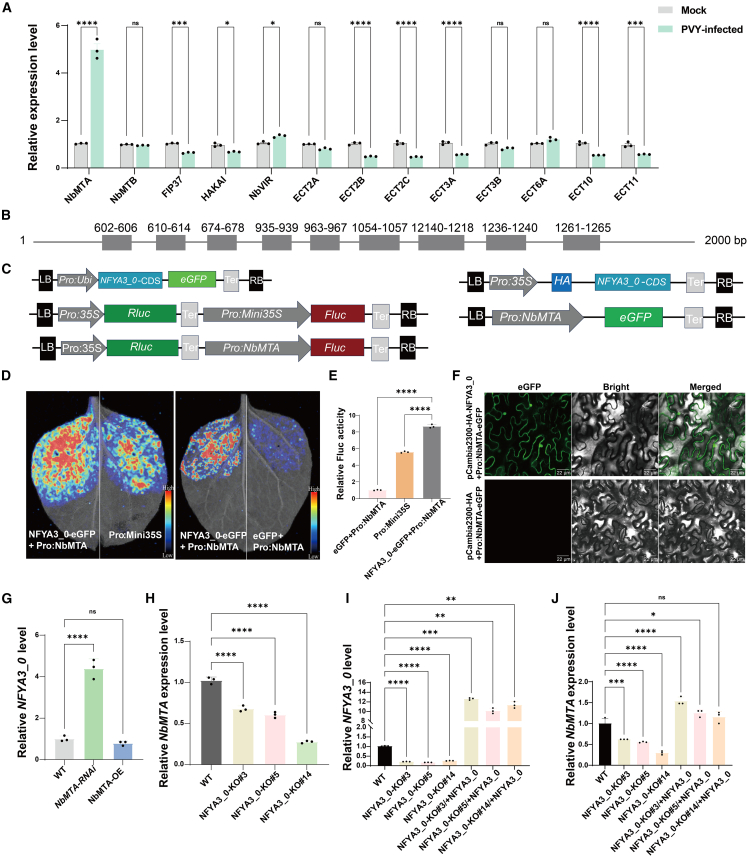
NFYA3_0 activates *NbMTA* transcription. **(A)** RT-qPCR analysis of the transcript levels of methyltransferase- and reader protein–related genes in healthy and PVY-infected plants. Asterisks indicate significant differences based on two-way ANOVA (*∗∗P* < 0.01; *∗∗∗P* < 0.001; *∗∗∗∗P* < 0.0001; ns, *P* > 0.05). Transcript-level comparisons between the two groups: *NbMTA* (*P* < 0.0001), *NbMTB* (*P* > 0.999), *FIP37* (*P* = 0.0006), *HAKAI* (*P* = 0.0161), *NbVIR* (*P* = 0.0130), *ECT2A* (*P* = 0.2045), *ECT2B* (*P* < 0.0001), *ECT2C* (*P* < 0.0001), *ECT3A* (*P* < 0.0001), *ECT3B* (*P* = 0.1439), *ECT6A* (*P* = 0.3562), *ECT10* (*P* < 0.0001), *ECT11* (*P* = 0.0004). Error bars represent SD (*n* = 3). **(B)** Map of the *NbMTA* promoter region containing the NF-Y family–specific binding motif CCAAT. **(C)** Schematic illustration of vector construction for investigating the regulatory relationship between NFYA3_0 and NbMTA. **(D)** Firefly luciferase activity detection results from transient expression assays in *N*. *benthamiana* leaves. **(E)** Dual-luciferase reporter gene activity corresponding to **(D)**. **(F)** Verification of NFYA3_0-activated *NbMTA* transcription using fluorescence confocal microscopy at 48 h post-inoculation. Scale bar: 22 μm. **(G)** RT-qPCR detection of relative *NFYA3_0* expression levels in WT, *NbMTA-OE*, and *NbMTA-RNAi* plants. **(H)** RT-qPCR analysis of NbMTA transcript levels in *NFYA3_0* KO plants. **(I)** Relative transcript levels of *NFYA3_0* in *NFYA3_0* KO plants after transient overexpression of *NFYA3_0* via *Agrobacterium* infiltration. WT and *NFYA3_0 KO* lines served as controls. **(J)** Relative transcript levels of *NbMTA* in *NFYA3_0* KO plants after transient overexpression of *NFYA3_0* via *Agrobacterium* infiltration (NFYA3_0-KO/+NFYA3_0). WT and *NFYA3_0* KO lines served as controls. In **(E)** and **(G)–(J)**, asterisks indicate significant differences based on one-way ANOVA (*∗P* < 0.05; *∗∗P* < 0.01; *∗∗∗P* < 0.001; *∗∗∗∗P* < 0.0001; ns, *P* > 0.05). Error bars represent SD (*n* = 3).

We used RT-qPCR to conduct systematic expression profiling of m^6^A methyltransferase complex components and reader protein–encoding genes to elucidate the core epigenetic regulators responsible for m^6^A hypermodification triggered by early viral infection. The results revealed that expression of the core methyltransferase gene *NbMTA* was upregulated by 4.99-fold, whereas reader genes (e.g., *ECT2A/B/C*) were substantially downregulated. These changes established a causal link between early m^6^A elevation and subsequent methyltransferase activation, collectively suggesting a pivotal role for *NbMTA* in antiviral responses (Figure 4A). Bioinformatics analysis identified nine CCAAT box motifs in the *NbMTA* promoter region (Figure 4B), echoing the overall m^6^A reduction observed in *NFYA3_0*-deficient plants. Thus, we performed a dual-luciferase reporter assay and found that *NFYA3_0* overexpression enhanced *NbMTA* promoter activity by 8.68-fold (*P* < 0.0001) (Figures 4D and 4E). This transcriptional activation was confirmed using promoter–EGFP fusion constructs; confocal microscopy showed significantly intensified EGFP fluorescence upon NFYA3_0 co-expression (Figure 4F). This result validated NFYA3_0’s regulatory role at the transcriptional and translational levels.

Gene expression analysis revealed that *NbMTA* overexpression did not affect *NFYA3_0* expression compared with the WT (*P* = 0.5898, one-way analysis of variance [ANOVA]), whereas *NFYA3_0* expression was significantly elevated in *NbMTA* knockdown lines (*P* < 0.0001, one-way ANOVA) (Figure 4G). Conversely, *NFYA3_0* KO reduced *NbMTA* expression compared with the WT (*P* < 0.0001, one-way ANOVA) (Figure 4H), a defect completely rescued by *NFYA3_0* complementation (Figures 4I and 4J). Genetic evidence supported a feedback loop between NFYA3_0 and NbMTA, in which NFYA3_0 regulates *NbMTA* transcription and, in turn, NbMTA regulates NFYA3_0 through m^6^A modification of its mRNA.

### NbMTA enhances plant antiviral immunity by regulating m^6^A methylation

**Figure 5:**
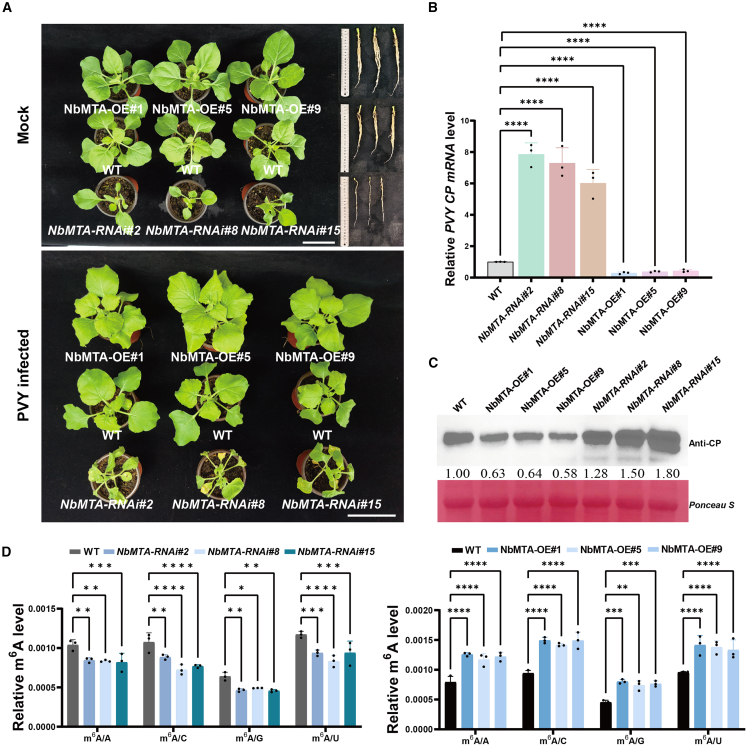
NbMTA enhances plant antiviral immunity by regulating m^6^A methylation. **(A)** Diagram of phenotypes in *NbMTA*-overexpressing and *NbMTA-*knockdown *N. benthamiana* plants under healthy and PVY-infected conditions. Scale bars: 10 cm. **(B)** RT-qPCR detection of relative PVY *CP* expression. Asterisks indicate significant differences based on one-way ANOVA (*∗∗∗∗P* < 0.0001). **(C)** Western blot analysis of PVY CP protein content corresponding to **(A)**. **(D)** LC–MS/MS detection of changes in mRNA m^6^A levels in plants after *NbMTA* overexpression or knockdown. Asterisks indicate significant differences based on two-way ANOVA (*∗P* < 0.01; *∗∗P* < 0.01; *∗∗∗P* < 0.001; *∗∗∗∗P* < 0.0001). Error bars represent SD (*n* = 3).

A TRV-VIGS vector was engineered to specifically downregulate *NbMTA* expression to systematically investigate the biological function of NbMTA in antiviral defense (silencing efficiency validation shown in Supplemental Figure 9B). Notably, *NbMTA*-deficient plants displayed significantly aggravated leaf wrinkling symptoms at 7 dpi (Supplemental Figure 9A). Viral quantification analyses revealed that PVY RNA expression in silenced plants increased by 3.06-fold compared with TRV-GUS controls, accompanied by a 2.73-fold increase in viral protein accumulation, collectively demonstrating that *NbMTA* depletion greatly enhances host susceptibility (Supplemental Figures 9C and 9D). Because CRISPR-Cas9–mediated KO of *NbMTA* resulted in embryonic lethality (consistent with previous reports) (Zhong et al., 2008), we generated a stable transgenic line overexpressing *NbMTA* (*NbMTA-OE*) and an *NbMTA* RNAi line (*NbMTA-RNAi*) (Supplemental Figures 10A and 10B). Phenotypical analysis revealed that *NbMTA-OE* plants developed normally, whereas *NbMTA-RNAi* plants exhibited considerable dwarfism (Figure 5A). PVY infection assays indicated that *NbMTA-OE* plants generally showed milder symptoms and had an average viral load reduction of 63% compared with the WT; *NbMTA-RNAi* plants accumulated 7.06-fold more viral RNA (*P* < 0.0001, one-way ANOVA) and 1.53-fold more viral protein (Figure 5B and 5C). LC–MS/MS quantification confirmed that global m^6^A modification levels were significantly increased in *NbMTA-OE* plants and decreased in *NbMTA-RNAi* plants compared with WT controls, a trend consistently observed in both total RNA and mRNA analyses (*P* < 0.05, two-way ANOVA) (Figure 5D and Supplemental Figure 11). This complementary genetic evidence definitively established that NbMTA positively regulates plant antiviral immunity through modulation of m^6^A methylation.

After establishing that NbMTA confers specific resistance against PVY via the m^6^A methylation pathway, we investigated whether its antiviral activity exhibits broad-spectrum functionality. To this end, we selected three taxonomically distinct viruses with divergent genomic structures—cucumber mosaic virus, cucumber green mottle mosaic virus, and pepper mild mottle virus—and used these to inoculate WT, *NbMTA-OE*, and *NbMTA-RNAi* plants. The results demonstrated that NbMTA clearly mediates broad-spectrum antiviral resistance. *NbMTA-OE* plants exhibited strong resistance against all three viruses, with significantly diminished disease symptoms (Supplemental Figures 12A, 12D, and 12G), as well as greatly reduced viral RNA accumulation (Supplemental Figures 12B, 12E, and 12H) and protein expression levels (Supplemental Figures 12C, 12F, and 12I). In contrast, *NbMTA-RNAi* plants showed enhanced susceptibility to all viruses tested, supporting significantly higher viral replication levels than the WT controls. These findings indicate that NbMTA-mediated m^6^A methylation is a key mechanism underlying basal antiviral immunity in plants, conferring broad-spectrum resistance against viruses from multiple genera and providing an important theoretical foundation for targeting *NbMTA* when engineering broad-spectrum antiviral crops.

### NbMTA enhances antiviral immunity through m^6^A-mediated viral RNA methylation and degradation

**Figure 6:**
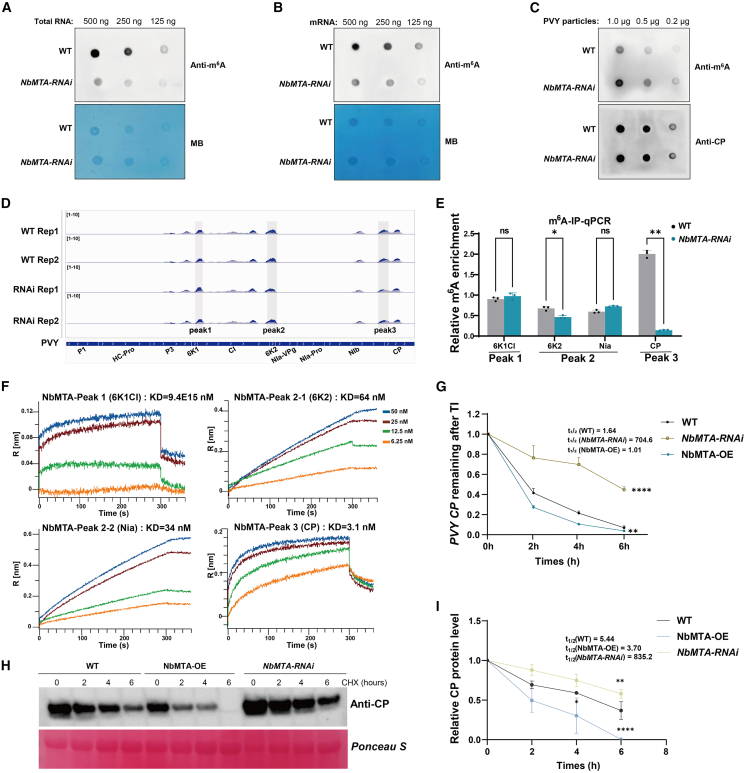
NbMTA exerts its function via m^6^A-mediated viral RNA methylation and degradation. **(A)** m^6^A dot blot analysis of total RNA from PVY-infected WT and *NbMTA-RNAi* plants; 0.1% methylene blue staining was used for nucleic acid quantification. **(B)** m^6^A dot blot analysis of mRNA from PVY-infected WT and *NbMTA-RNAi* plants; 0.1% methylene blue staining was used for nucleic acid quantification. **(C)** Dot blot detection of m^6^A modification levels in viral particles purified from PVY-infected WT and *NbMTA*-knockdown plants. Equal loading was verified with an anti-CP antibody. **(D)** Identification of m^6^A methylation peaks in the PVY genome from WT and *NbMTA-RNAi plants* using MeRIP-seq; gray represents input (front) and dark blue represents IP (rear). **(E)** m6A IP–qPCR targeting CP mRNA in *NbMTA* lines. Asterisks indicate significant differences based on two-way ANOVA (*∗P* < 0.05; *∗∗P* < 0.01; ns, *P* > 0.05). *6K1CI* (*P* = 0.8323), *6K2* (*P* = 0.0489), *Nia* (*P* = 0.0530), *CP* (*P* = 0.0022). **(F)** BLI detection of binding affinity between RNA and NbMTA protein across differentially methylated regions. **(G)** Stability analysis of CP mRNA in WT, *NbMTA-OE*, and *NbMTA-RNAi N*. *benthamiana* after actinomycin D treatment. Shown are relative CP mRNA levels over time. **(H)** Western blot analysis of viral protein levels in WT, *NbMTA-OE*, and *NbMTA-RNAi* plants treated with cycloheximide at the indicated time points. Ponceau S staining was used to confirm equal protein loading. **(I)** Quantification of viral protein levels from three independent protein stability assays, used to evaluate viral protein stability in WT, *NbMTA-OE*, and *NbMTA-RNAi* plants. In **(E)**, **(I)**, and **(G)**, asterisks indicate significant differences based on two-way ANOVA (*∗∗P* < 0.01; *∗∗∗∗P* < 0.0001). Error bars represent SD (*n* = 3).

Available evidence indicates that host-driven m^6^A hypermodification of viral RNA serves as a potent restriction factor against pathogen proliferation (He et al., 2023, He et al., 2024; Sha et al., 2024). Therefore, we assessed the global impact of *NbMTA* depletion on m^6^A modification to determine whether NbMTA exerts its antiviral function through direct epitranscriptomic editing of viral RNA. An m^6^A-specific antibody dot blot assay revealed that global m^6^A modification levels in total RNA and mRNA were significantly lower in PVY-infected *NbMTA-RNAi* plants than in WT plants (Figures 6A and 6B). Furthermore, to elucidate the relationship between viral RNA accumulation and m^6^A methylation, PVY virions were purified from WT and *NbMTA-RNAi* plants. Dot blot analysis showed a significantly reduced m^6^A methylation level in viral RNA derived from *NbMTA-RNAi* plants compared with that from WT plants (Figure 6C). These results collectively establish a central role for NbMTA in mediating m^6^A modification of viral RNA.

We performed MeRIP-seq analyses on both plant total mRNA and viral RNA from PVY-infected WT and *NbMTA-RNAi* plants to dissect this role at global and site-specific levels and to assess its impact on the host transcriptome. At the host level, MeRIP-seq analysis showed that *NbMTA* knockdown resulted in a significant reduction of m^6^A peaks within 144 host mRNAs; only 75 peaks displayed a significant increase (*P* < 0.05) (Supplemental Figure 13A). Notably, GO enrichment analysis indicated that these hypomethylated genes were significantly enriched in pathways such as “negative regulation of mRNA decay,” “mRNA stability,” and “negative regulation of RNA metabolic process” (Supplemental Figure 13B), suggesting that *NbMTA* deficiency disrupts the stability and metabolic balance of host mRNAs. At the virus level, we identified three distinct NbMTA-dependent m^6^A modification sites in the PVY genome. Among these, the most prominent modified peak (peak 3) was located within the *CP* coding region, and its intensity was reduced by 15.4% in knockdown plants (*P* < 0.0001, Mann–Whitney U test; Figure 6D). Site-specific hypomethylation at this locus was independently validated using m^6^A IP–qPCR (*P* = 0.0022, two-way ANOVA; Figure 6E). These results demonstrate that NbMTA restricts viral proliferation by modulating host RNA homeostasis and by directly hypermodifying key viral RNA sites, such as the *CP* coding region.

We performed biolayer interferometry (BLI) using synthetic single-stranded RNA probes targeting differential peak regions to mechanistically investigate NbMTA–viral RNA interactions. NbMTA exhibited high-affinity binding to *CP* regions (K_D_ = 3.1 μM), weaker binding to 6K2 and NIa regions (K_D_ > 30 μM), and no specific binding to 6K1CI regions (Figure 6F). Kinetic profiling of viral RNA decay revealed diametrically opposed stability landscapes. *NbMTA-OE* plants exhibited accelerated RNA degradation (half-life [t_1/2_]: *NbMTA-OE* = 1.01 h; WT = 1.64 h; *P* = 0.0021 vs. WT), whereas *NbMTA-RNAi* plants displayed prominent transcript persistence (t_1/2_: *NbMTA-RNAi* = 704.6 h; *P* < 0.0001 vs. WT), as determined by first-order decay modeling (Figure 6G). Moreover, viral protein stability assays consistently indicated that viral proteins were highly unstable in *NbMTA-OE* plants (t_1/2_ = 3.70 h, *P* < 0.0001 vs. WT) but were robustly stabilized in *NbMTA-RNAi* plants (t_1/2_ = 835.2 h; t_1/2_ [WT] = 5.44 h, *P* = 0.0028 vs. WT).

These results demonstrated an inverse relationship between m^6^A-dependent RNA decay efficiency and viral replication capability. In this study, we identified NbMTA as a key m^6^A writer enzyme that specifically anchors to and methylates the PVY *CP* coding region, thereby promoting rapid viral RNA degradation via host surveillance machinery and effectively reducing systemic infection.

## Discussion

**Figure 7:**
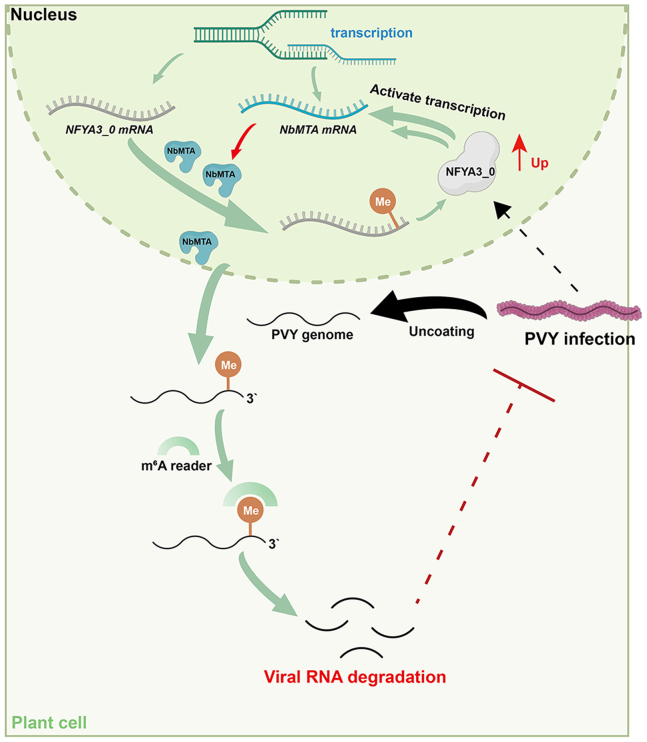
Molecular mechanism by which plants employ the NFYA3_0–NbMTA–m^6^A cascade to defend against viral invasion. Proposed working model showing that NFYA3_0 activates *NbMTA* to modify viral RNA methylation and promote viral RNA degradation, thereby influencing PVY infection.

This study elucidates a sophisticated antiviral defense axis in plants, centered on the NFYA3_0–NbMTA–m^6^A cascade, which operates through a tripartite mechanism to counteract viral invasion (Figure 7). At the core of this pathway lies a bidirectional positive feedback loop. As a master transcriptional regulator, NFYA3_0 coordinates m^6^A homeostasis by directly activating *NbMTA* expression. In turn, NbMTA installs m^6^A modifications on NFYA3_0 mRNA and enhances its expression via m^6^A-mediated mRNA stabilization, thus amplifying the antiviral signal. Consequently, NbMTA serves as a key effector in antiviral surveillance. Its antiviral function is executed through a three-tiered molecular strategy: (1) sequence-specific recognition and binding of PVY RNA through its catalytic domain, (2) site-directed m^6^A methylation at the *CP* coding region, and (3) activation of m^6^A-dependent RNA decay pathways. The m^6^A modification serves as a molecular “barcode” to trigger the host RNA surveillance mechanism, which recognizes methylated viral RNA as an aberrant transcript for direct clearance, reducing viral replication efficiency and suppressing systemic infection. This report constitutes the first demonstration in plants of a vertically integrated antiviral pathway linking transcriptional regulation to epitranscriptomic editing, thereby fundamentally reshaping the paradigm of plant–pathogen interactions.

Plants have evolved a complex immune system for surveillance, perception, and defense activation during long-term evolution (Li et al., 2020). RNA epigenetic modifications, particularly the dynamic m^6^A methylation system, play pivotal roles in plant stress responses. This system precisely regulates RNA metabolism (stability, translation, and splicing) to coordinate expression reprogramming of stress-responsive genes (Ferraz et al., 2022; Li et al., 2025). For example, the *Arabidopsis* methyltransferase AtFIP37 enhances disease resistance by mediating m^6^A modification of *PR* gene mRNAs, whereas rice OsMTA2 participates in immune responses upon viral infection, highlighting the crucial role of m^6^A modification in plant–pathogen interactions (Arribas-Hernández and Brodersen, 2019; Hu et al., 2019; He et al., 2024). However, compared with mammalian systems, studies of the functional mechanisms of plant RNA methyltransferases in biotic stress responses—particularly viral defense—remain limited, especially regarding specific roles in combating biological threats.

The discovery of the NFYA3_0–NbMTA–m^6^A signaling cascade elucidates a previously unrecognized layer of antiviral defense in plants, integrating transcriptional regulation, epigenetic modification, and RNA metabolism into a cohesive tripartite mechanism. This study builds upon emerging evidence of the role of m^6^A methylation in plant–virus interactions, in which viral RNAs can be methylated and targeted for degradation by host machinery (Yue et al., 2022a, 2022b). For instance, host m^6^A modifications dynamically shift during PVY invasion, and ALKB homologs mediate demethylation to stabilize viral genomes (Yue et al., 2022a). In contrast, our findings reveal a proactive host strategy whereby NbMTA selectively methylates viral RNAs to trigger their decay, establishing a direct link between m^6^A deposition and antiviral immunity. This mechanistic divergence underscores the complexity of m^6^A’s dual role in plant–virus coevolution: hosts exploit it for defense, whereas viruses may subvert it for survival.

The identification of NFYA3_0 as an upstream regulator of *NbMTA* transcription expands the broader understanding of how plants coordinate transcriptional and epigenetic responses to pathogens. Transcription factors such as NFYA3_0 modulate stress-responsive gene networks; however, their involvement in epigenetic reprogramming during antiviral defense has not been previously defined. This phenomenon mirrors analogous systems in animals, where YTHDF2 recognizes m^6^A modifications in the 5′ UTR of the E26 transformation-specific variant transcription factor 5 mRNA and recruits eukaryotic translation initiation factor 3 subunit B to facilitate its translation (Wen et al., 2024). Notably, the NFYA3_0–NbMTA axis maintains host m^6^A homeostasis by counteracting virus-induced hypomethylation, a phenomenon observed in PVY-infected plants (Yue et al., 2022a). By activating *NbMTA* expression, NFYA3_0 ensures sustained antiviral activity, underscoring the critical role of transcriptional fine-tuning in epigenetic defense strategies.

The conserved function of MTA as the catalytic core of the m^6^A methyltransferase complex has been validated across plant species. Our observation that *NbMTA* overexpression elevates global m^6^A levels is consistent with previous reports. Loss-of-function *mta* mutants in *Arabidopsis thaliana* exhibit severely reduced m^6^A accumulation (Bodi et al., 2012), whereas *MTA* overexpression enhances m^6^A modification of specific targets such as primary microRNAs (Bhat et al., 2020; Wang et al., 2023). Similarly, *NbMTA* overexpression in *N*. *benthamiana* increases m^6^A deposition on viral RNA and confers enhanced antiviral resistance (He et al., 2024). The moderate but important global increase in m^6^A levels (∼1.19-fold) observed in the present study aligns with the anticipated biological scope of MTA activity, which is naturally constrained *in vivo* by the availability of partner subunits and S-adenosylmethionine cofactors. Thus, our results strongly reinforce the notion that NbMTA serves as a central conserved regulator of m^6^A methylation. Furthermore, the specific deployment of this core catalytic subunit through direct transcriptional induction by NFYA3_0 reveals a previously unrecognized regulatory layer underlying antiviral epitranscriptomic responses.

In plant–virus coevolution, the host regulates RNA metabolism through m^6^A-dependent RNA decay mechanisms such as NMD (He et al., 2024; Tang et al., 2023). In the present study, we found that NbMTA increased the degradation efficiency of viral RNA by more than 60% via binding and methylation of viral RNA, as well as activation of the decay pathway. Its deletion led to abnormally stable viral transcripts (t_1/2_ > 700 h) (Figure 6G), confirming a central role for m^6^A dynamic modification in antiviral immunity (He et al., 2023; Martínez-Pérez et al., 2023; Li et al., 2025). This represents a functional distinction from the observation that TaMTB in wheat enhances viral RNA stability through m^6^A modification (Zhang et al., 2022), suggesting that the effect of the host m^6^A system depends on evolutionary dynamics; it can either label viral RNA for degradation (e.g., NbMTA) or be exploited by the virus to maintain stability (e.g., TaMTB). This bidirectional regulation offers new avenues for antiviral strategies; such as enhancing host m^6^A methylation through CRISPR–dCas13 or related technologies to target viral RNA degradation (Yu et al., 2024), or regulating demethylases to balance resistance and yield (Jia et al., 2011), thereby providing targets for epitranscriptome-based disease-resistance breeding.

Future studies could investigate whether the NFYA3_0–NbMTA–m^6^A regulatory module is functional in other plant–pathogen systems, including potential interactions with innate immune components such as NLR proteins. It would also be valuable to examine whether m^6^A methylation exerts selective pressure on viral genomes, potentially influencing viral evolution through the targeting of methylated regions. Notably, a study in *Phytophthora sojae* indicated that m^6^A affects pathogen virulence and DNA damage repair (Seidl et al., 2024), suggesting that m^6^A can also contribute to plant virus adaptation. From a translational perspective, our results imply that methods to increase *NbMTA* or *NFYA3_0* expression—for instance, through genetic engineering—may offer a strategy for developing crop varieties with enhanced resistance to multiple plant viruses.

## Methods

### Plant culture and inoculation with PVY

WT, *NbMTA-OE*, *NbMTA-RNAi*, and *NFYA3_0-KO* *N*. *benthamiana* seedlings were grown in an artificial climate maintained at 24°C, 50% relative humidity, and a photoperiod of 16 h light/8 h darkness. The PVY source was maintained on *Nicotiana tabacum* L. and preserved by the Fine Chemical Research and Development Center of Guizhou University. To prepare the inoculum, 1.0 g of virus-infected tissue was ground in liquid nitrogen. Subsequently, 5 ml of precooled virus extraction buffer (0.665 g Na_2_HPO_4_·12H_2_O, 6.6 g NaH_2_PO_4_·2H_2_O, 1.26 g Na_2_SO_3_, and 1 l double distilled water, pH 7.0) was added and homogenized thoroughly. Emery powder was evenly applied to the two basal leaves, and the virus extract was gently rubbed onto *N*. *benthamiana* leaves with a row pen. The inoculated leaves were rinsed after 30 min, and seedlings were transferred to a greenhouse.

### Vector construction

Full-length coding sequences of *NbMTA* and *NFYA3_0*, as well as specific fragments of 250–300 bp, were amplified using cDNA derived from total RNA extracted from *N*. *benthamiana* and gene-specific primers. Genomic DNA from *N*. *benthamiana* served as the template to amplify *proNbMTA* by PCR. PCR products were resolved on a 1.0% agarose gel to confirm expected band sizes, then subjected to gel extraction and purification. Recombinant plasmids were generated by inserting PCR-amplified fragments into linearized vectors after double digestion, using the ClonExpress II One Step Cloning Kit (Vazyme, C112-01) per the manufacturer’s instructions. The resulting plasmids were pBWA(V)HS-NbMTA-3×FLAG, pRTV-NFYA3_0-EGFP, pCambia2300-HA-NFYA3_0, pGreenII-0800-ProNbMTA, pGreenII-0800-proNbMTA-EGFP, TRV-NbMTA, and TRV-NFYA3_0. The recombinant products were transformed into DH5*α*-competent cells via heat shock. Single colonies were selected and confirmed by bidirectional sequencing, and the constructed plasmids were stored at −20°C for subsequent use. Detailed information regarding the DNA primers used for vector assembly is provided in Table S1.

### Extraction of mRNA from *N*. *benthamiana*

Total RNA was isolated from *N*. *benthamiana* seedlings (100 mg fresh tissue per replicate). Polyadenylated mRNA was enriched using Dynabeads Oligo(dT)_25_ (Thermo Fisher Scientific, 61006), in accordance with the manufacturer’s protocol; two consecutive rounds of purification were conducted to deplete rRNA contamination. The typical mRNA yield from *N*. *benthamiana* total RNA was approximately 0.5% (w/w). Purified mRNA was quantified using the Qubit RNA High Sensitivity Assay Kit (Thermo Fisher Scientific, Q32852) and stored at −80°C for downstream applications.

### LC–MS/MS detection of m^6^A levels in PVY-infected *N*. *benthamiana*

Nuclease P1 (1 U) (Wako Pure Chemical Industries) and 10% 0.1 M ammonium acetate (pH 5.3) were added to 200 ng mRNA and mixed well. The mixture was incubated at 42°C for 3.0 h to digest the mRNA into single nucleotides. Subsequently, 1 U of recombinant Shrimp Alkaline Phosphatase (New England Biolabs) and 10% CutSmart buffer (New England Biolabs) were added and incubated at 37°C for 3–4 h to remove phosphate groups. The processed sample was centrifuged at 20 625 *g* for 30 min; LC–MS/MS (Agilent Technologies, AB SCIEX) was then used to detect the levels of various nucleosides. Cations were selected for detection in multiple reaction monitoring mode, with parent and daughter ion *m/z* values of 268.0/136.0 (A), 282.0/150.1 (m^6^A), 244.0/112.0 (C), 284.0/152.0 (G), and 245.0/113.1 (U), respectively. A series of nucleoside standards at known concentrations was used to construct a calibration curve, and sample values were interpolated on this curve to determine nucleoside concentrations for quantitative analysis.

### Instantaneous conversion of *N*. *benthamiana*

A single, correctly transformed GV3101 colony was inoculated into 5 ml of Luria-Bertani liquid medium containing kanamycin (50 mg l^−1^) and rifampicin (25 mg l^−1^), then incubated at 28°C with shaking at 220 rpm until the culture reached an optical density at 600 nm (OD_600_) of 1.0–2.0. The bacterial solution was diluted 1:200 into 20 ml of Luria-Bertani liquid medium containing the above antibiotics and shaken until the OD_600_ reached approximately 1.0. Cells were collected by centrifugation at 2,817 *g* for 10 min, and the resulting pellet was resuspended in infiltration solution (10 mM MgCl_2_, 10 mM 2-morpholinoethanesulfonic acid [pH 5.6]) containing 100 μM acetosyringone. The OD_600_ was adjusted to 0.6–0.8, and the suspension was incubated for 2.0 h at room temperature. *Agrobacterium* suspensions were then infiltrated into the abaxial epidermis of four fully expanded leaves per *N*. *benthamiana* plant (leaf area ≈ 4.0 cm^2^) using a needleless syringe.

### RNA-seq

Total RNA was extracted from plant leaves using TRIzol reagent (Invitrogen). Poly(A)+ RNA was subsequently isolated from the total RNA using oligo(dT)_25_ Dynabeads (Thermo Fisher Scientific), in accordance with the manufacturer’s instructions. RNA fragmentation reagents (New England Biolabs) were then used to fragment the poly(A)+ RNA into ∼100-nt fragments. Library construction was performed using the NEBNext Ultra II RNA Library Preparation Kit (New England Biolabs), and sequencing was conducted in paired-end mode on an Illumina HiSeq X Ten platform with 150 bp reads (Genewiz) (Song et al., 2021).

### m^6^A sequencing

Total RNA and 5.0 μg poly(A)+ RNA were extracted from *N*. *benthamiana* leaves. RNA fragmentation reagents (New England Biolabs) were then used to fragment the poly(A)+ RNA into ∼100-nt fragments, and 50.0 ng of fragmented poly(A)+ RNA was used as input for RNA-seq. The remaining mRNA samples were processed according to the *N*^6^-Methyladenosine Enrichment Kit (New England Biolabs) manual to enrich m^6A-^containing fragments. Poly(A)+ RNA from the input and poly(A)+ RNA from IP were used to construct libraries with the NEBNext Ultra II RNA Library Preparation Kit (New England Biolabs). Sequencing was conducted in paired-end mode on an Illumina HiSeq X Ten platform with 150 bp reads (Genewiz).

### RNA-seq data analysis

Sequencing reads were trimmed using Cutadapt (v1.18) and mapped to the Nbe1.01 reference genome using HISAT2 (v2.1.0). SAMTOOLS v1.9 was used for sequence alignment/map-to-binary alignment/map conversion and sorting. Fragments per kilobase of exon per million mapped fragments (FPKM) values were calculated using StringTie (v1.3.5). Differentially expressed genes in PVY- and mock-treated plants were identified using the R package Ballgown, with criteria of FPKM fold change > 1.5 and *P* < 0.05.

### m^6^A sequencing data analysis

For m^6^A sequencing, reads ≥ 50 nt after trimming were selected and mapped to the Nbe1.01 reference genome using HISAT2 (v2.1.0). The MACS2 peak-calling algorithm was used to identify m^6A-enriched^ regions as m^6^A peaks (Cai et al., 2024).

### VIGS-mediated silencing of *NbMTA* and *NFYA3_0*

Bacterial suspensions of *Agrobacterium* harboring TRV-VIGS vectors (pTRV1 with pTRV2 derivatives: PDS, GUS, NbMTA, and NFYA3_0) were prepared in infiltration medium (OD_600_ = 1.0) and combined at equal volumes. After 2 h of incubation at room temperature, co-cultured strains were pressure infiltrated into fully expanded leaves of *N*. *benthamiana* plants at the fourth and fifth true leaf developmental stages. When the PDS silencing phenotype appeared in TRV-PDS–treated plants as a positive control, the silencing of *NbMTA* and *NFYA3_0* was confirmed, and the TRV-GUS– or TRV-VIGS–treated *N*. *benthamiana* plants were inoculated with PVY. Symptoms of viral infection, viral accumulation, and changes in m^6^A levels were then recorded.

### NFYA3_0-mediated activation of *NbMTA* transcription

The *Agrobacterium tumefaciens* (*A. tumefaciens)* strain harboring pGreenII-0800-ProNbMTA was adjusted to OD_600_ = 0.6 and mixed in equal volumes with *Agrobacterium* cultures (OD_600_ = 0.6) containing either pRTV-NFYA3_0-eGFP or pRTV-eGFP (empty vector) for the dual-luciferase reporter assay. The pGreenII-0800-ProMini35S construct served as the positive control. After 2 h of incubation in the dark at 25°C, bacterial mixtures were infiltrated into *N*. *benthamiana* leaves using consistent injection sites and volumes. At 48 h post-infiltration, luciferin sodium substrate (Sangon Biotech, Shanghai, China) was applied to the leaves for chemiluminescence imaging. Simultaneously, infiltrated leaf tissues were collected and analyzed using the Dual-Luciferase Reporter Assay Kit (YEASEN, Shanghai, China) to quantify firefly and *Renilla* luciferase activities.

Recombinant vectors containing ProNbMTA–EGFP fusion and hemagglutinin (HA)-tagged NFYA3_0 were transformed into *A. tumefaciens* strain GV3101 for the GFP reporter assay. After centrifugation at 2,817 *g* for 10 min, the bacterial pellets were resuspended in infiltration buffer and adjusted to OD_600_ = 0.6. Bacterial suspensions were mixed at a 1:1 ratio (ProNbMTA–EGFP:HA-NFYA3_0 or ProNbMTA:HA as a control) and incubated in darkness at 25°C for 2 h before co-infiltration into *N*. *benthamiana* leaves. At 48 h post-infiltration, fluorescence signals were visualized and recorded using a confocal laser scanning microscope (Nikon, Tokyo, Japan).

### mRNA stability assays

mRNA stability assays were performed using transcriptional inhibition by actinomycin D (Wei et al., 2018). Briefly, apical leaves from uniformly infected WT, *NbMTA-OE*, and *NbMTA-RNAi* plants were transferred into 20 ml of 1/2 Murashige and Skoog liquid medium. After 1 h of incubation, actinomycin D (Sigma-Aldrich) was added to the medium at a final concentration of 200 μM. Seedlings collected 1 h after infiltration served as the 0 h control, and subsequent samples were harvested every 2 h (Song et al., 2021). Two biological replicates were performed, each comprising approximately 10 plants per time point. RNA isolation and RT-qPCR analysis were conducted as described above to quantify mRNA levels; *Actin* mRNA served as a negative control. All primers are listed in Table S1.

### Biolayer interferometry (BLI) assays

Biotinylated single-stranded RNA (100 nM) was loaded onto Super Streptavidin biosensors, and signals of binding to NbMTA protein (50 nmol l^−1^) were measured using BLI with an Octet RED 96 system (ForteBio, San Francisco, CA, USA) (Zan et al., 2025). Samples underwent initial equilibration in 0.01 M saline before analysis. Sequential incubation cycles were performed on the biosensors, beginning with serially diluted NbMTA protein solutions, followed by phosphate-buffered saline washing to monitor dissociation kinetics. Quantification of RNA–protein binding affinity (K_D_ values) was conducted through BLI using a ForteBio Octet Discovery 12.2 platform. Experimental binding curves were computationally processed via global fitting algorithms implemented in ForteBio’s proprietary Octet Analysis Studio 12.2 software.

## Data and code availability

The MeRIP-seq datasets generated in this study have been deposited in the OMIX database at the China National Genomics Data Center under accession number OMIX012725 (related to project PRJCA049168). All other data supporting the findings of this study are available within the article and its supplemental information files.

## Funding

We acknowledge financial support from the 10.13039/501100012166National Key Research and Development Program of China (2022YFD1700300).

## Acknowledgments

We are grateful to Prof. Guifang Jia from the School of Chemistry and Molecular Engineering, Peking University, for providing technical support related to m^6^A modification. No conflict of interest is declared.

## Author contributions

J. Li, methodology, investigation, visualization, data curation, writing – original draft; J. Luo, investigation, writing – original draft; H.H., visualization, writing – original draft; F.W., methodology, writing – original draft; R.S., funding acquisition, project administration, resources, supervision, writing – review & editing; B.S., conceptualization, funding acquisition, project administration, resources, supervision, writing – review & editing.

## Supplemental information

Document S1. Supplemental Figures 1–13, Supplemental Tables 1–7, and Supplemental Methods 1

Supplemental Data 1. MeRIP-seq analysis data: m^6^A and Transcriptome in PVY-infected vs. mock, and in WT vs. *NbMTA-RNAi* Plants

Document S2. Article plus supplemental information
