# Supplementary material for: Transcription factor NFYA3_0 promotes MTA-mediated m6A modification of PVY genomic RNA to confer antiviral resistance in Nicotiana benthamiana
Source: Plant Commun. 2025 Oct 31;7(1):101584. doi: 10.1016/j.xplc.2025.101584 (PMC12902261; doi:10.1016/j.xplc.2025.101584)
Supplement: Document S1. Supplemental Figures 1–13, Supplemental Tables 1–7, and Supplemental Methods 1 [file mmc1.pdf]

Plant Communications, Volume *7*

Supplemental information

Transcription factor NFYA3_0 promotes MTA-mediated m^{6}A modification of PVY genomic RNA to confer antiviral resistance in *Nicotiana benthamiana*

Jiao Li, Jianli Luo, Hongfu He, Fenyan Wang, Huan Wu, Chunni Zhao, Runjiang Song, and Baoan Song

Supplemental Information

The nuclear transcription factor NFYA3_0 promotes MTA-mediated m6A modification of Potato virus Y genomic RNA to confer antiviral resistance in *Nicotiana benthamiana*

Jiao Li[1], Jianli Luo[1], Hongfu He[1], Fenyan Wang[1], Huan Wu[1], Chunni Zhao[1], Runjiang Song[1,*], Baoan Song[1,*]

[1]State Key Laboratory of Green Pesticide, Guizhou University, Guiyang, 550025, P.R. China.

[*]Correspondence: Baoan Song (basong@gzu.edu.cn) or Runjiang Song (songrj@gzu.edu.cn)

Supplemental Methods

Supplemental Figures 1-13

Supplemental Tables S1-7

Supplemental Methods

Recombinant protein expression and purification

The cDNA of *N. benthamiana* was used as a template, the CDS of NbMTA gene was amplified by PCR, and the size of the target band was confirmed by running 1% Agarose gel and the PCR product was purified and recovered. The PCR-amplified CDS sequence was inserted into the vector pCold^{TM} I using the ClonExpress II One Step Cloning Kit (Vazyme) as described and named pCold^{TM} I-NbMTA. The recombinant product was transformed into DH5*α* competent cells by heat shock method, monoclonal was selected and double-ended sequencing for detection, and the constructed plasmid was transformed into *E. coli* BL21 Gold (DE3) competent cells. Monoclonal was picked into 10 mL of LB containing 50 *μ*g/mL ampicillin and cultured overnight at 37°C at 200 rpm. The OD_{600} value was measured after shaking 10 mL of saturated *E. coli* into 1 L LB containing 50 *μ*g/mL ampicillin at 37°C, 180 rpm, and shaking for 2.5 h. The OD_{600} value for controlling *E. coli* is between 0.6-0.8. Add 500 μM IPTG and induce protein expression at 16°C for 14-16 h. Centrifugation at 6,000 rpm for 15 min, 40 mL of lysate (10 mM imidazole, pH 7.4, 10 mM Tris, 500 mM NaCl, 1 mM PMSF, 5% glycerol, 4 M urea) was resuspended, and ultrasonic lysis on ice was resuspended for 45 minutes (power 20 W, 5 s on, 5 s off). Centrifugation at 13,000 rpm for 30 minutes, 0.45 μm membrane supernatant filtration. The filtered fragmented solution was loaded onto a Ni-NTA column (GE Healthcare) that had been balanced with buffer A (10 mM imidazole, pH 7.4, 10 mM Tris, 150 mM NaCl, 10% glycerol, 4 M urea) at a flow rate of 1 mL/min, and rinsed with buffer A for 30 mL at a flow rate of 1 mL/min to baseline equilibration. Samples were eluted with elution buffer B (500 mM imidazole, pH 7.4, 10 mM Tris, 150 mM NaCl, 10% glycerol, 4 M urea) and the protein solution was collected. The protein solution was centrifuged at 3,000 rpm at 4°C and concentrated to 2-5 mL, during which the buffer was continuously changed to

remove the urea from the protein solution to allow the purpose to be refolded. The resulting protein solution was centrifuged at 12,000 rpm for 15 min, the supernatant was transferred to a small centrifuge tube, and non-denaturing SDS-PAGE electrophoresis and denaturing SDS-PAGE electrophoresis were performed to determine the target protein solution. Finally, the protein solution was aliquoted into 0.2 mL tubes per 10 μL tube and stored at -80°C after liquid nitrogen flash freezing.

qRT-PCR identification of target gene expression levels

According to the instructions of qPCR Master Mix (Yeasen), relative quantitative analysis of the gene expression level of the target protein was performed using *Actin* as an internal reference gene. Generally, 1.0 *μ*L of cDNA diluted twice is added as a template for a single reaction, and 0.8 *μ*L of forward and reverse primers (concentration 10 *μ*M) are used each.

m^{6}A-IP-qPCR:

m^{6}A-IP quantitative PCR (qPCR) was performed as previously reported. In short, random hexamers (R223-01; Vazyme, Nanjing, China) were used to input RNA and immunoprecipitated RNA. Measure relative mRNA enrichment using quantitative reverse transcriptase (qRT)-PCR and normalize to input level. The primers used are listed in Supplemental Table S1. Incubate 5.0 *μ*g of fragmented poly (A)+ RNA with 5.0 *μ*g of m^{6}A antibody (Synaptic Systems) and immunoprecipitated using pre blocked protein A Dynabeads (Thermo Fisher Scientific). The RNA and Input of m^{6}A-IP immunoprecipitation were reverse transcribed, and the enrichment factor of specific transcripts was calculated by qPCR measurement. *Actin* is used as an internal control gene.

m^6A dot blot:

Total RNA or mRNA serially diluted was denatured at 95°C for 3 minutes to disrupt secondary structures, and then immediately cooled on ice to prevent reassociation. 2 *μ*L of the denatured RNA samples were spotted onto a Hybond-N+ membrane optimized for nucleic acid transfer, followed by cross-linking under UV light at 254 nm for 5 minutes. The membrane was then blocked with 5% non-fat milk in TBST buffer for 1 hour at room temperature with gentle shaking and washed three times gently with 1× TBST buffer. Subsequently, the membrane was incubated overnight at 4°C with anti-m^6A antibody (Cat No. 68055-1-Ig, Proteintech®, USA) diluted 1:2000. After incubation, the membrane was washed three times with 1×TBST, followed by incubation with HRP-conjugated secondary antibody diluted 1:5000 in 10 mL of 1×TBST for 1 hour at room temperature with gentle shaking. The membrane was then washed again three times with 1×TBST. Finally, Clarity Western ECL Substrate was applied, and the signal was detected using a chemiluminescence imaging system (ChemiDoc, Bio-Rad, USA).

Protein stability experiments：

Plant leaves with uniform growth status were selected and equilibrated in 1/2 MS buffer for 30 minutes. A portion of the sample was collected as the 0-hour control, while the remainder was transferred to a culture dish or centrifuge tube containing 100 mM cycloheximide (CHX) working solution and incubated with gentle shaking. Sampling was performed at 2, 4, and 6 hours after CHX addition. At each time point, an equal amount of material was quickly retrieved, excess liquid was removed, and the samples were immediately frozen in liquid nitrogen to terminate all biochemical reactions. The samples were ground into a fine powder under liquid nitrogen protection. Pre-chilled protein extraction buffer

containing protease inhibitors was added immediately. The mixture was kept on ice for 30 minutes with intermittent vortexing to facilitate dissolution. Centrifugation was carried out at 4°C and 12,000 × g for 15 minutes. The supernatant (total protein extract) was carefully transferred to a new pre-chilled tube, avoiding any pellet. The extracted proteins were either quantified immediately or aliquoted and stored at -80°C. Subsequent analysis was performed by Western Blotting. Nonlinear regression fitting and half-life calculations were conducted using GraphPad Prism software.

Supplemental Figures

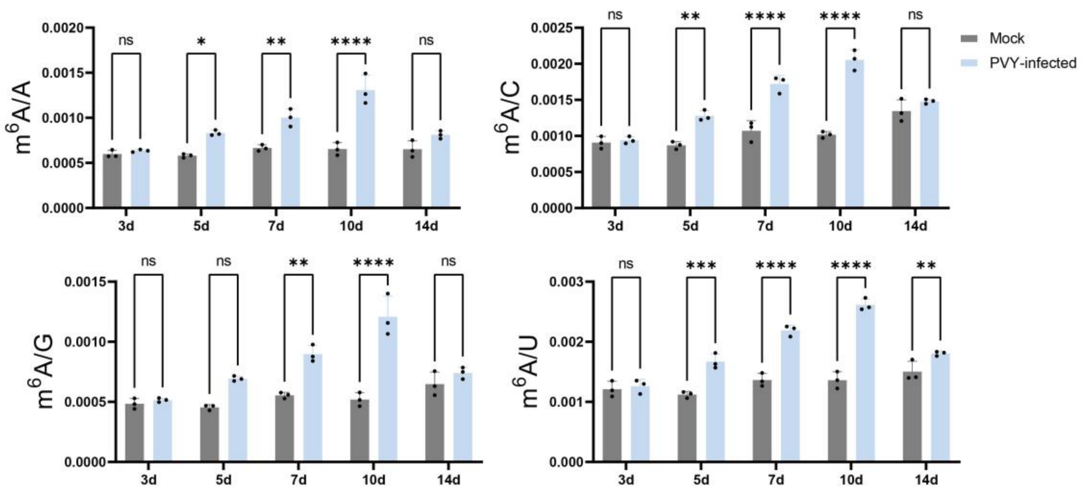

Supplemental Figure 1. LC-MS/MS analysis of the global m[6]A modification level in total RNA from Mock and PVY-infected *N. benthamiana* plants.

The asterisk indicated a significant difference between Mock and PVY-infected *N. benthamiana* based on the two-way ANOVA analysis (***, P* < 0.01; ****, P* < 0.001). Error bars indicate standard deviation (*n* = 3).

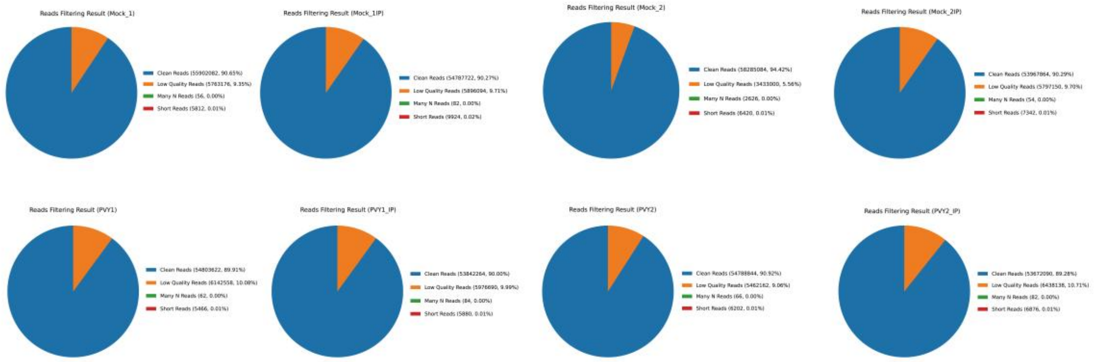

Supplemental Figure 2. Library data preprocessing and analysis.

Raw data filtering, FASTP (version 0.23.1) software is used for data quality control, removing adapter sequences in reads, and filtering out reads with a length of less than 18BP; Filter low-quality reads (when the proportion of bases with a quality value lower than Q20 is greater than 0.08); Filter the reads that read N (when the reads read N more than 5 bases).

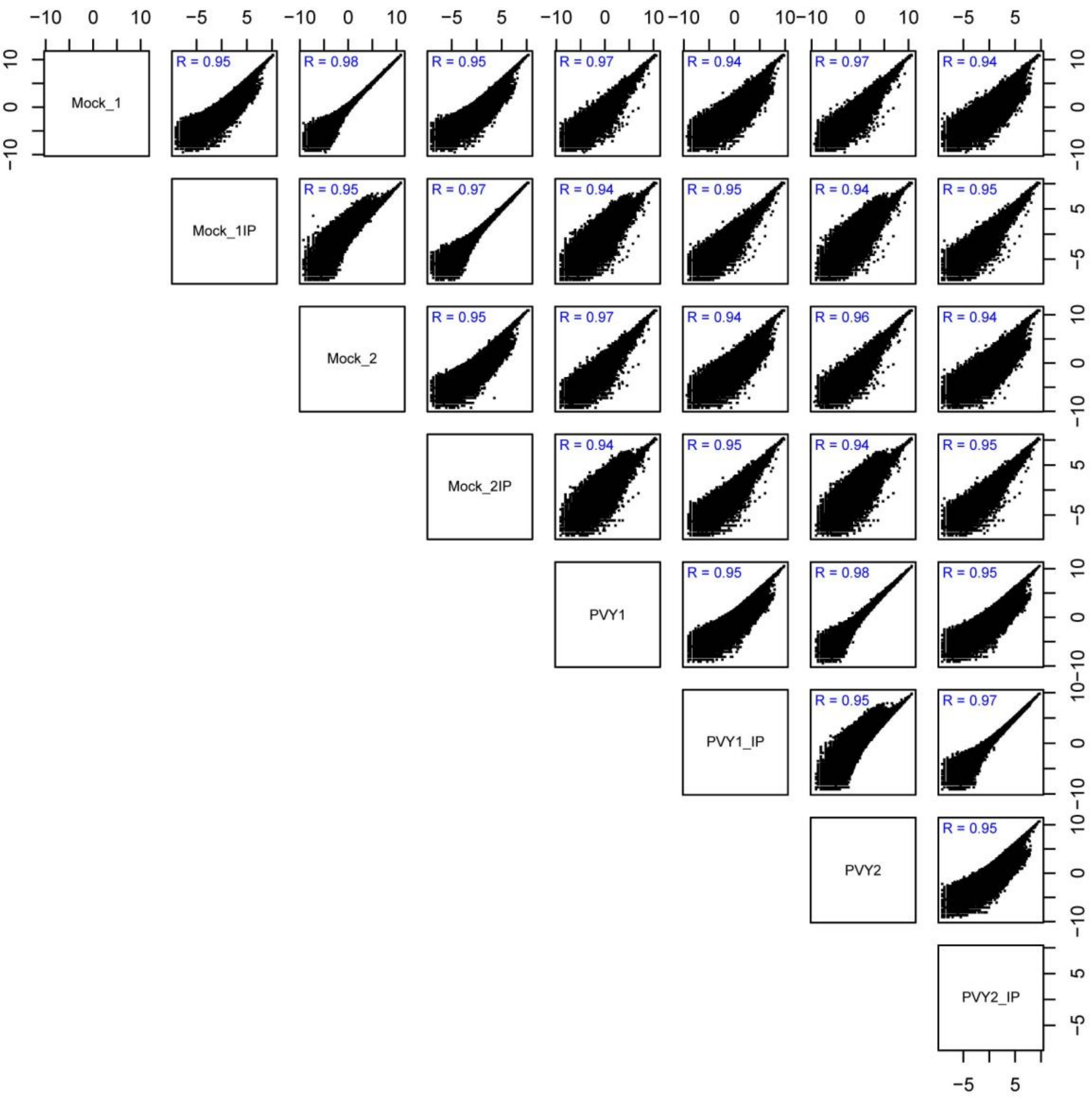

Supplemental Figure 3. Scatter plot of gene correlation between samples.

The abscissa and ordinate represent $\log_2$(reads count) on each exon, respectively. The scatters are biased to the side of the IP group, indicating that IP group specific enrichment of methylated mRNA.

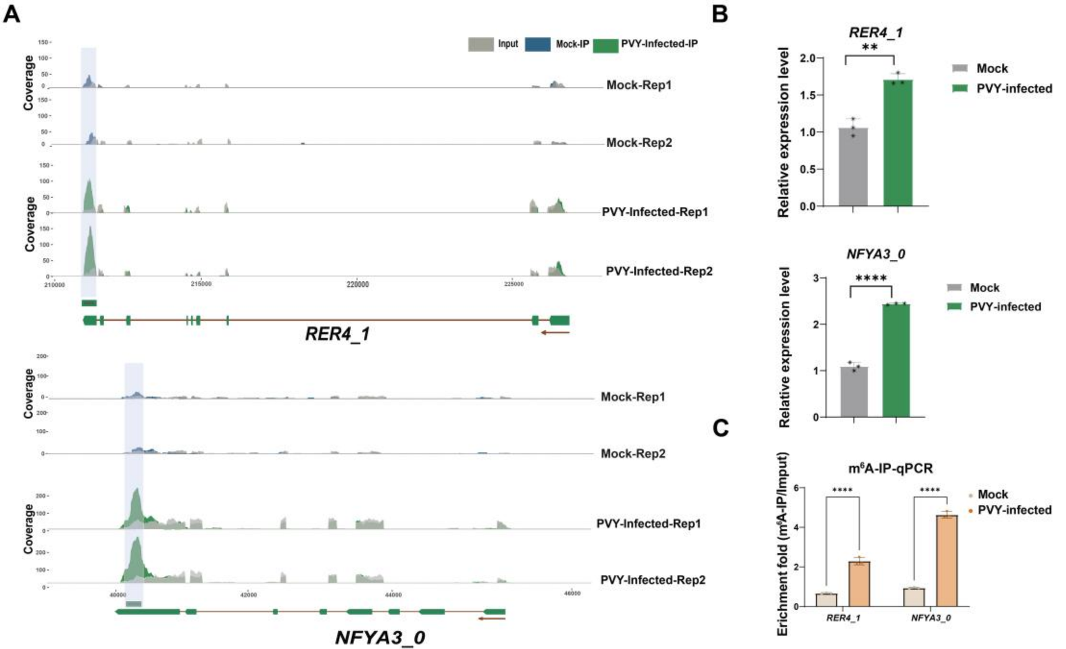

Supplemental Figure 4. Verify the reliability of MeRIP-seq data.

(A) Visualization of the distribution of m^{6}A reads in m^{6}A peak transcripts of *N. benthamiana* plants under healthy and PVY infection conditions in the Integrated Genome Viewer. Gray indicates input reads. Blue and green represent immunoprecipitation readings. The direction of the arrow indicates the direction of gene transcription, and the thick box and thick line represent exons and introns, respectively.

(B) qRT-PCR was used to detect the relative expression levels of transcripts in *N. benthamiana* plants under healthy and PVY-infected conditions, and the asterisk indicated a significant difference between healthy and PVY-infected *N. benthamiana* based on *T*-test (bilateral) (**, $P < 0.01$; ***, $P < 0.001$). Error bars indicate the standard deviation ($n = 3$).

(C) m^{6}A-IP-qPCR validation results of peaks in RER4_1 and NFYA3_0. The gene *Actin* is used as an internal control gene. The asterisk indicated a significant difference between healthy and PVY-infected *N. benthamiana* based on the two-way ANOVA analysis (**, $P < 0.01$; ***, $P < 0.001$). Error bars indicate standard deviation ($n = 3$).

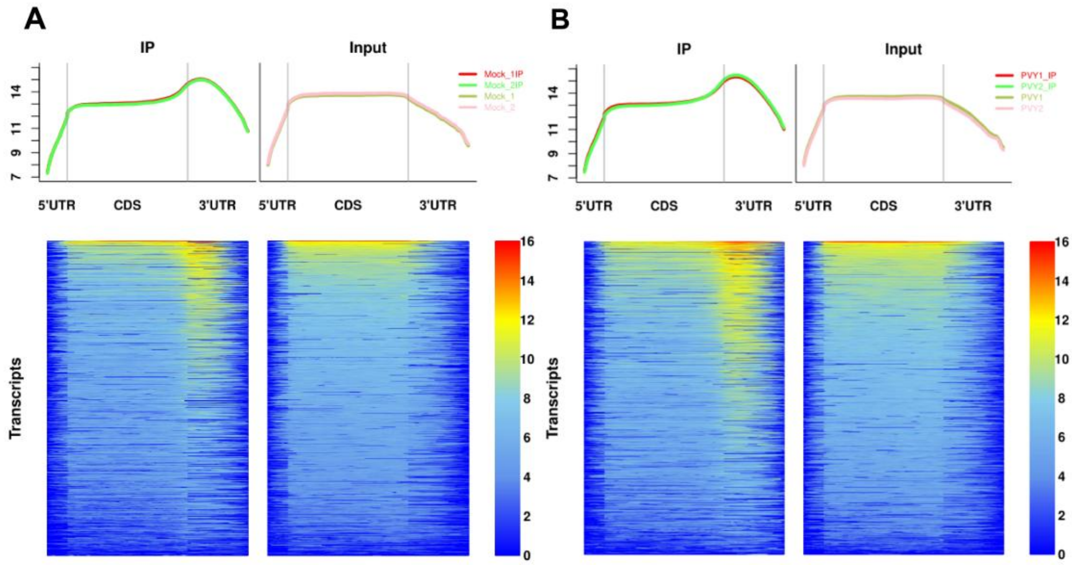

Supplemental Figure 5. Reads are distributed across the functional regions of the peak associated genes.

The coverage of reads on the genes annotated by peak was counted, and the distribution map (and heat map) of reads on the functional region of the peak gene was made. The left and right graphs represent the distribution of reads in the IP and Input samples across all the gene functional regions to which the peak is annotated. The above figure shows the cumulative distribution of reads on all gene functional regions (the total reads are taken as the logarithm of 10), and the bottom figure shows the distribution of reads on each gene, with the color gradient from blue to yellow to red, representing the coverage depth from shallow to dark.

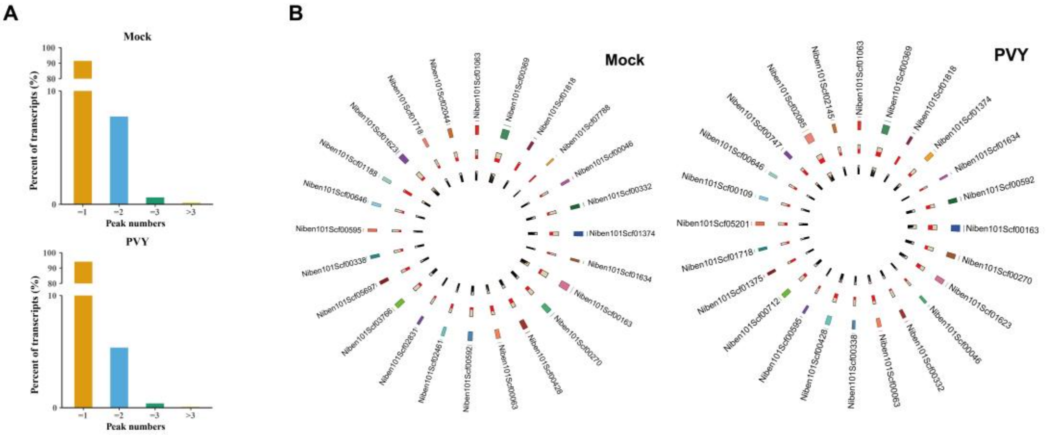

Supplemental Figure 6. Statistics on the number of m^{6}A peaks on each transcript and distribution statistics on genomic scaffolds.

(A) Statistical plot of m^{6}A peaks for each transcript.

(B) Distribution of m^{6}A peaks is plotted along scaffold coordinates. The outermost circle is the length abbreviation of the scaffold, the inner circle is the m^{6}A peak number distribution on each scaffold, and the innermost circle is the m^{6}A peak enrichment fold distribution on each scaffold.

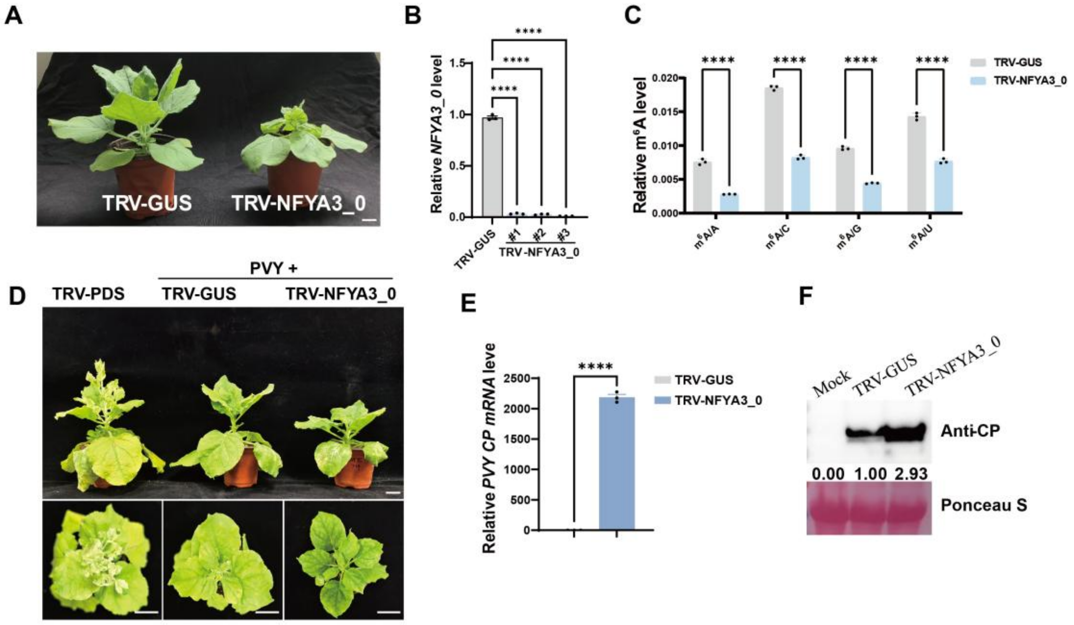

Supplemental Figure 7. The results of VIGS showed that *NFYA3_0* was involved in the m^{6}A modification pathway and inhibited viral invasion.

(A) Diagram of plant symptoms of TRV-GUS and TRV-NFYA3_0. Bar = 5 cm.

(B) Detection of the silencing efficiency of gene *NFYA3_0*. Asterisks indicate significant differences in sample content based on one-way ANOVA analysis (****, P < 0.0001).

(C) LC-MS/MS was used to detect the m^{6}A level of plants after gene *NFYA3_0* silencing. Asterisks indicate significant differences in sample content based on two-way ANOVA analysis (****, P < 0.0001).

(D) Symptom map of day 7 of PVY inoculation after gene *NFYA3_0* silencing. Bar = 5 cm.

(E) qRT-PCR was used to detect the relative expression level of PVY CP in (D). The asterisk indicates a significant difference in virus content based on *T*-test (bilateral) analysis (****, P < 0.001).

(F) Western blot analysis of PVY CP protein content in **(**D).

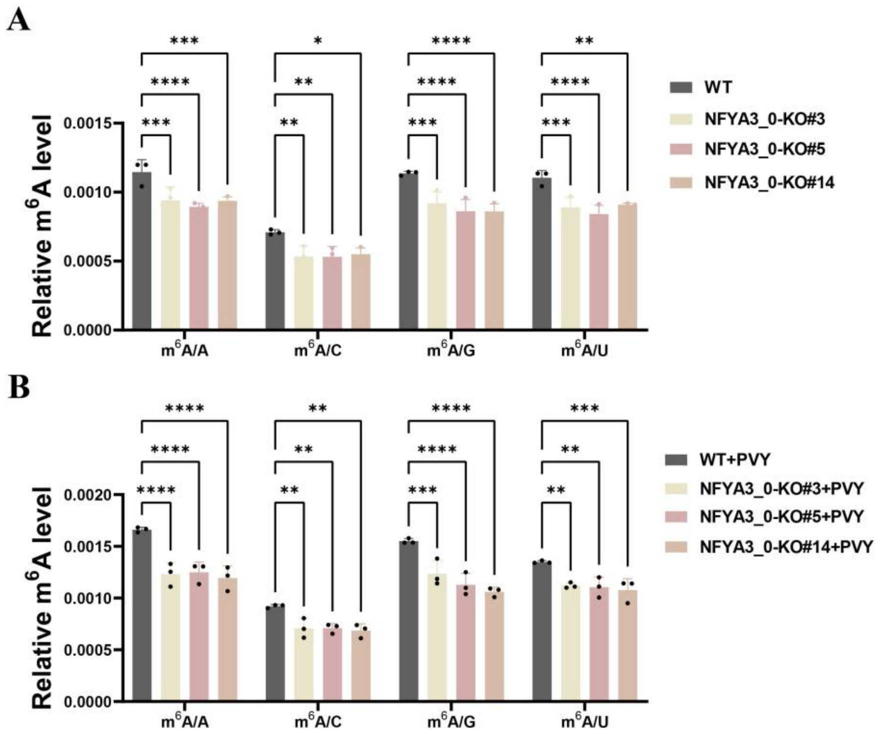

Supplemental Figure 8. LC-MS/MS analysis of global m^{6}A modification levels in total RNA from WT and various *NFYA3_0* knockout lines under healthy and PVY-infected conditions.

(**A**) LC-MS/MS analysis of global m^{6}A modification levels in total RNA from WT and various *NFYA3_0* knockout lines under healthy conditions. Asterisks indicate significant differences in sample content based on two-way ANOVA analysis (*, $P < 0.05$; **, $P < 0.01$; ****, $P < 0.0001$). Error bars indicate standard deviation ($n = 3$).

(**B**) LC-MS/MS analysis of global m^{6}A modification levels in total RNA from WT and various *NFYA3_0* knockout lines under PVY-infected conditions. Asterisks indicate significant differences in sample content based on two-way ANOVA analysis (*, $P < 0.05$; **, $P < 0.01$; ****, $P < 0.0001$). Error bars indicate standard deviation ($n = 3$).

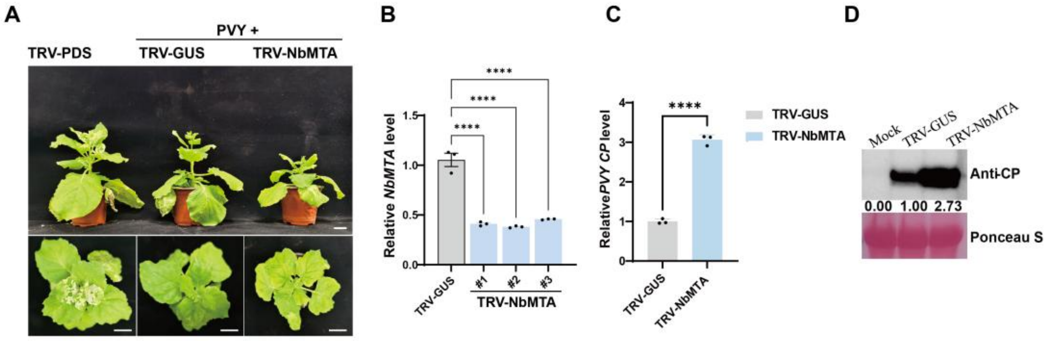

Supplemental Figure 9. VIGS-mediated *NbMTA* silencing in *N. benthamiana* reduced plant antiviral immunity.

(A) Symptom plot on day 7 of PVY inoculation after gene *NbMTA* silencing. Bar = 5 cm.

(B) Detection of the silencing efficiency of the gene *NbMTA* (****, $P < 0.0001$, one-way ANOVA).

(C) qRT-PCR was used to detect the relative expression level of *PVY CP* in (A) (****, $P < 0.0001$, *T*-test-based (bilateral)).

(D) Western blot analysis of PVY CP protein content in (A).

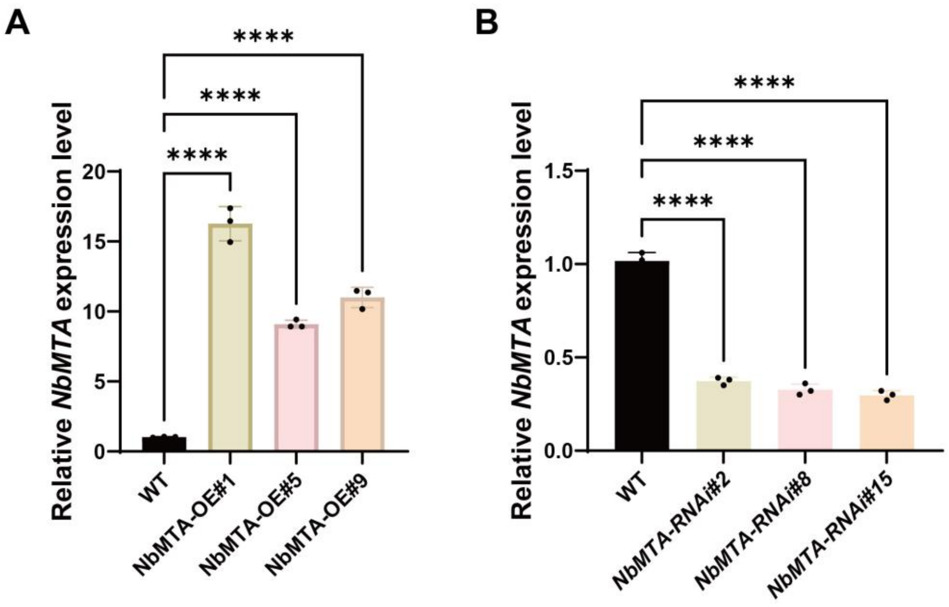

Supplemental Figure 10. Confirm overexpression and knockdown of *NbMTA*.

(A) qRT-PCR detection of relative expression levels of *NbMTA* in plants transformed into *NbMTA* overexpression vector, with an asterisk indicating a significant difference between WT and *NbMTA* overexpressing *N. benthamiana* based on one-way ANOVA (****, P < 0.0001). Error bars indicate the standard deviation (n = 3).

(B) qRT-PCR detection of relative expression levels of *NbMTA* in plants transformed into *NbMTA* RNAi vector, with an asterisk indicating a significant difference between WT and *NbMTA* overexpressing *N. benthamiana* based on one-way ANOVA (****, P < 0.0001). Error bars indicate the standard deviation (n = 3).

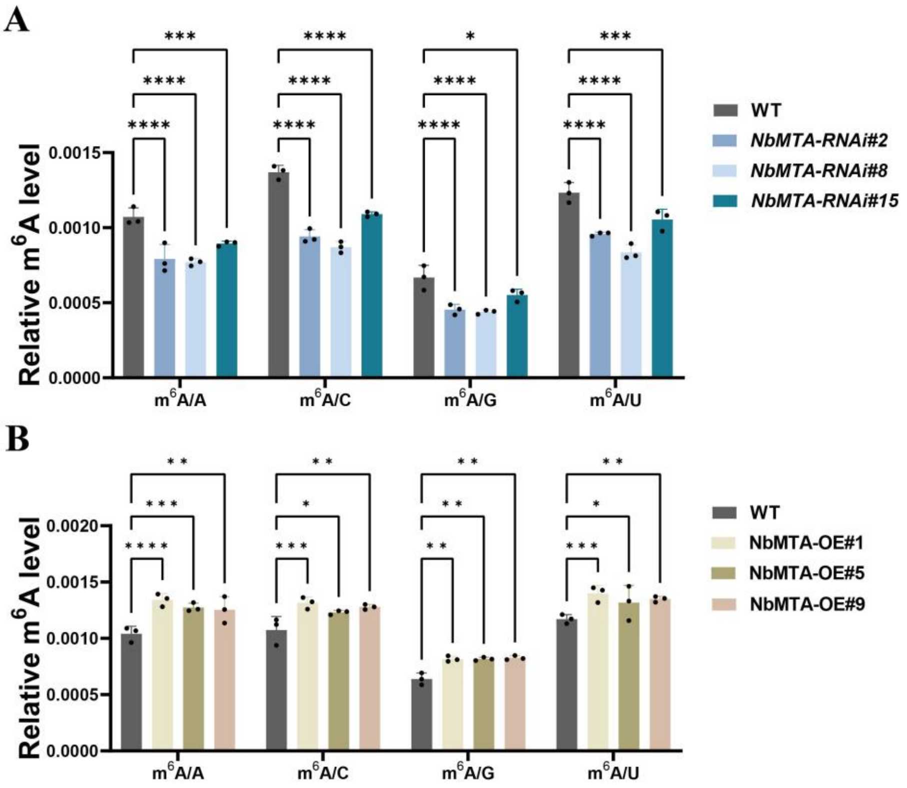

Supplemental Figure 11. The changes in total RNA m^{6}A levels were detected by LC-MS/MS in plants after overexpression and knockdown of *NbMTA*.

(A) The changes in total RNA m^{6}A levels were detected by LC-MS/MS in plants after knockdown of *NbMTA*. Asterisks indicate significant differences between samples based on two-way ANOVA analysis (*, $P < 0.01$; **, $P < 0.01$; ***, $P < 0.001$; ****, $P < 0.0001$). Error bars indicate the standard deviation ($n = 3$).

(B) The changes in total RNA m^{6}A levels were detected by LC-MS/MS in plants after overexpression. Asterisks indicate significant differences between samples based on two-way ANOVA analysis (*, $P < 0.01$; **, $P < 0.01$; ***, $P < 0.001$; ****, $P < 0.0001$). Error bars indicate the standard deviation ($n = 3$).

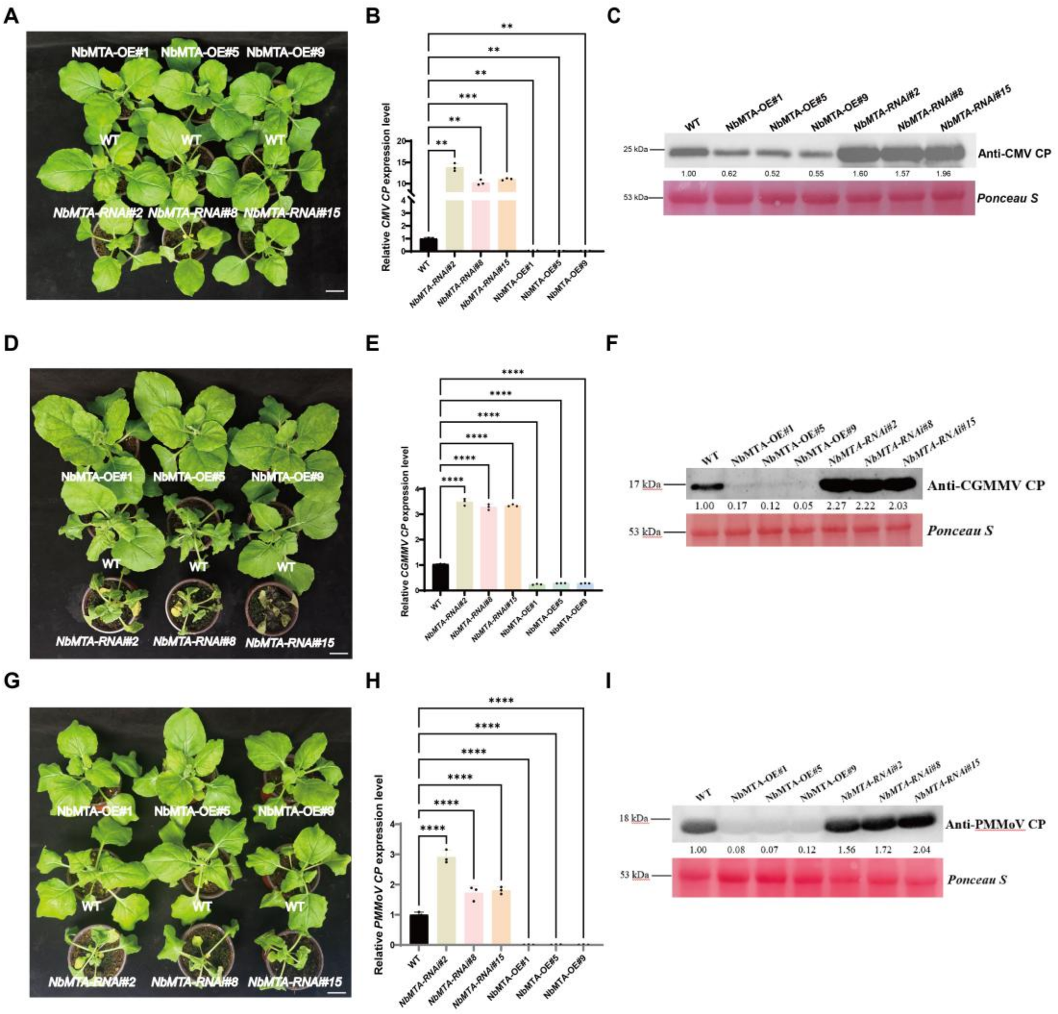

Supplemental Figure 12. Investigation of the broad-spectrum antiviral activity of NbMTA against plant viruses.

(**A**), (**D**), and (**G**) Phenotypes of WT, NbMTA-OE, and *NbMTA-RNAi* plants inoculated with CMV, CGMMV, and PMMoV, respectively. Bar = 6 cm.

(**B**), (**E**), and (**H**) Viral RNA accumulation levels detected by RT-qPCR in WT, NbMTA-OE, and *NbMTA-RNAi* plants after inoculation with CMV, CGMMV, and PMMoV, respectively. Asterisks indicate significant differences compared with WT plants as determined by one-way ANOVA (****, $P < 0.0001$). Error bars represent standard deviation ($n = 3$).

(**C**), (**F**), and (**I**) Viral protein accumulation levels detected by western blot in WT, NbMTA-OE, and *NbMTA-RNAi* plants inoculated with CMV, CGMMV, and PMMoV, respectively. *Ponceau S* staining was used as a loading control.

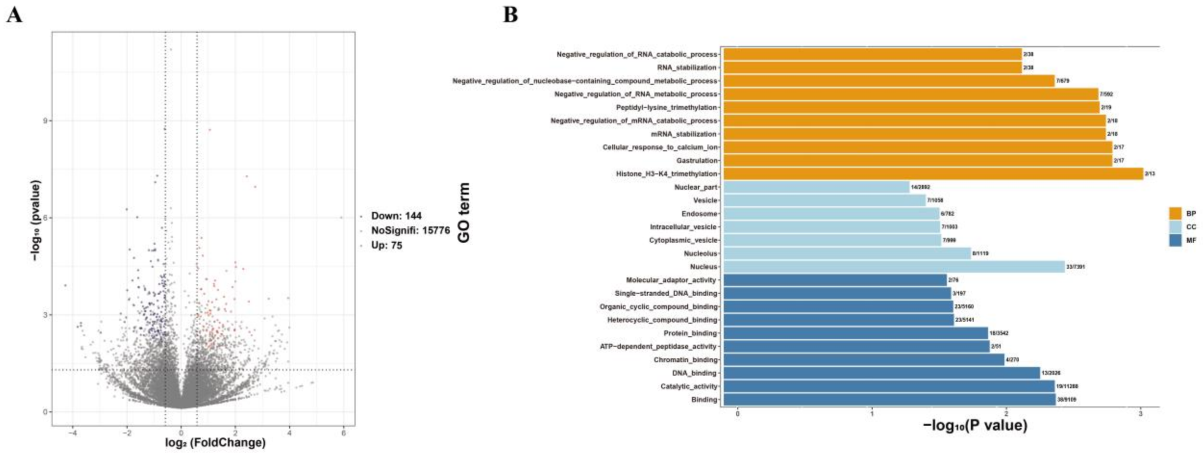

Supplemental Figure 13. MeRIP-seq analysis was performed on total mRNA extracted from PVY-infected WT and *NbMTA-RNAi* plants.

(**A**) Volcano plot displaying differentially m^6A-methylated sites in PVY-infected WT versus *NbMTA-RNAi* plants. Differentially methylated sites were identified with a threshold of $|log_2 FoldChange| \geq 0.58$ and $P < 0.05$.

(**B**) GO enrichment analysis of genes with significantly hypomethylated m^6A sites.

Supplemental Tables

Table S1. List of oligonucleotides used in this study. Sequences are shown in 5`→3` direction

Primer name	Primer sequence (5' to 3')	Used for
PCR-BWA	TTTATCATCATCATCTTTATAATCAATGTCGTGGTCT	
PCR-BWA	TGTAACTAGCTCTGTCTTCAGTACTGGG	**Overexpression**
BWA-NbMTA-F	AAGATGATGATGATAAAATGGAAACTCACTCGGACG	
BWA-NbMTA-R	TGAAGACAGAGCTAGTTACATTAGCTTGCCATCTCC	
NFYA3_0-RTV-	ATCCAGATCCAGTGGGATCCATGATAAGCTTCTCCC	
NFYA3_0-RTV-	GCGGCCGCACTAGTAAGCTTTCAAGTTCCAACATG	**LUC**
proNbMTA1500-	TTGATATCGAATTCCTGCAGTGTGTTTTGTATATTCT	
proNbMTA1500-	GCTCTAGAACTAGTGGATCCTGTTGATATTTCTTTCA	
NFYA3_0-ip-q1-	TCATCTCCAACGCACGACAA	
NFYA3_0-ip-q1-	CTGAACTCTGGCTGCTGGAA	**m^6A-IP-qPCR**
RER4-ip-q1-F	TGGGTTGATTATGCTCGTTTGATC	
RER4-ip-q1-R	TGTACCTATGACTCAACTTCCTGA	
NbMTA-TRV-F	TTACCGAATTCTCTAGACACATGGCTGATGATGAAA	
NbMTA-TRV-R	AGCTCGGTACCGGATCCTGCCTGGATTTCCCTTTAT	**VIGS**
TRV-NFYA3_0-	GTTACCGAATTCTCTAGACACTTTATGAGGTGCAAG	
TRV-NFYA3_0-	AGCTCGGTACCGGATCCTGCCGGACTTGACGTCTT	
PVY CP-F	GGAAATGACACAATCGATGCAGG	**Virus detection**
PVY CP-R	TCCACCCATGTTCTTCACTCC	
proNbMTA-	ATATCAACAGGATCCATGGTGAGCAAGGGCGAG	
proNbMTA-	CGCTCTAGAACTAGTTTACTTGTACAGCTCGTCCAT	
1300-HA-KZ-R	CTGCAGGTCGACCTAAGCGTAATCTGGAACGTCGT	**GFP reporter**
1300-HA-R	GAGAAGCTTATCATGGATCCAGCGTAATCTGGAAC	
1300-NFYA3_0-	TTCCAGATTACGCTGGATCCATGATAAGCTTCTCCC	
1300-NFYA3_0-	TCTGCAGGTCGACCTAAGTTCCAACATGCATGAAGT	
NFYA3_0-q-F	TCATCTCCAACGCACGACAA	
NFYA3_0-q-R	GTGCCCCAACTACCACTCTC	
RER4_1-q-F	CGTCAACTTCCATCCGGTGA	**qPCR**
RER4_1-q-R	CTGCTGCCTCCATTACCTCC	
q-NbMTA-F	CCCCTTCCTAACACCCCTAA	

q-NbMTA-R	TCATTGCCCTTCCCATTCCC
PVY-CP-q-F	TCGGGCAACTCAATCACAGT
PVY-CP-q-R	GACTCCGTTGACATTTGGCG
q-NbActin-F	TCCACGAGACTACATACAAC
q-NbActin-R	GCCACCACCTTAATCTTCA
q-NbMTB-F	AGTTACCCGAAACAGGACGAC
q-NbMTB-R	GCCAGACCTTGGATTACTTGAC
q-NbFIP37-F	GGAGTCTTTCATACCACCTGGC
q-NbFIP37-R	GCTCTTTCCAACTGTTCACGC
q-NbHAKAI-F	AGCCTTGGTTCAGCAAATACTG
q-NbHAKAI-R	GACTTGAGACAATGCGGAGC
q-NbVIR-F	AACTTTCGTCCACCCGCAAC
q-NbVIR-R	TGAAGAGCAAGCAGAAGCCG
q-NbECT2A-F	TATCCCTCGTACTATTATGGGTATGATG
q-NbECT2A-R	TGATACATAAATGACCCGTTGTCC
q-NbECT2B-F	CCCAATAAATTTGGGAACACATTC
q-NbECT2B-R	ACCTGTTGTCAACCGGCG
q-NbECT2C-F	GTTATCCCTCTACTGCAGCATACTATTATG
q-NbECT2C-R	TGATACATAAATGACCCGTTGTCC
q-NbECT3A-F	GCGGTTTCACCTTCTGTTGC
q-NbECT3A-R	TTGGTTCGCTGAGTCACGG
q-NbECT3B-F	AGCAATACCAATACCCATCGTC
q-NbECT3B-R	GAAGGTGAAACCGCTGTCTG
q-NbECT6A-F	GATTTATTGGTCTGGATGGTCAAGA
q-NbECT6A-R	CAGGAATGTAAGGGTTGTATGGG
q-NbECT10-F	TCAGCCAGGCATTATTCCC
q-NbECT10-R	CGTCTTCCACCTCTGTCAGG
q-NbECT11-F	TTGCGATTACTACTACCCAGGC
q-NbECT11-R	GGGAACCATTATCTGATTGAATACC

Table S2. Sequence information of synthesized biotin single-stranded RNA

Name	Sequence Information
**Peak 1 (6K1CI)**	Bio-ctttcctcaatggaccatgaagttagacatcagtccttagacgatgtgatcaagaattttgatgagaggaatgagattattgattttgaattgagtgaggacacaattcgaacatcatcagtgctagatacaaagtttagtgattggtggg
**Peak 2-1 (6K2)**	Bio-tgcagttcgttcatcaccaagctacgacgtcacttgcaaaggatctcaagttgaaggggacttggaagaagtcattagtggccaaagacttgatcatagcaggcgctgttgcaattggtggaatagg actcatatatagttggttcacacaatcagttgagactgtgtctcaccaa
**Peak 2-1 (Nia)**	Bio-gggaaaaataaatccaaaagaattcaagccttgaagtttcgccatgctcgtgacaaaagggctggttttgaaattgacaacaatgatgacacaatagaggaattctttggatctgcatacaggaaaaagggaaaaggtaaaggtaccactgttggtatgggcaagtcaagcagaaggtttgttaa
**Peak 3 (CP)**	Bio-tctgggacacatactgtgccgagaatcaaggctatcacgtccaaaatgagaatgcccaaaagcaagggagcaaccgtgctaaacttagaacacttgcttgagtatgctccacaacaaattgatatttcaaatactcgggcaactcaatcaca

Table S3. Summary of raw MeRIP-seq data

Sample	Raw Reads	Raw Bases(G)	Raw Q20(%)	Raw Q30(%)	Raw GC(%)
**Mock_1**	61671126	9.25	97.02	92.25	46.09
**Mock_1IP**	60693822	9.10	96.62	91.63	44.29
**Mock_2**	61727130	9.26	98.21	95.05	45.59
**Mock_2IP**	59772410	8.97	95.68	90.63	44.65
**PVY1**	60951708	9.14	96.91	91.97	44.96
**PVY1_IP**	59824918	8.97	97.08	92.28	42.82
**PVY2**	60257274	9.04	96.74	91.86	46.46
**PVY2_IP**	60117186	9.02	96.97	91.98	42.56

* Sample: Sample Name; Raw Reads: Number of reads in raw downstream data; Raw Bases (G): Total number of bases in raw downstream data; Raw Q20 (%): Proportion of bases with quality greater than Q20 in raw downstream data; Raw Q30 (%): Proportion of bases with quality greater than Q30 in raw downstream data; Raw GC (%): Average GC content of bases in raw downstream data.

Table S4. Comprehensive quality control data table for MeRIP-seq

Sample	Clean Reads	Clean Bases(G)	Clean Q20(%)	Clean Q30(%)	Clean GC(%)	Effective Rate(%)
**Mock_1**	55902082	6.88	98.96	95.72	44.72	90.65
**Mock_1IP**	54787722	6.44	98.97	95.72	42.06	90.27
**Mock_2**	58285084	7.52	99.43	97.30	44.50	94.42
**Mock_2IP**	53967864	6.17	98.97	95.70	42.23	90.29
**PVY1**	54803622	7.02	98.85	95.36	43.72	89.91
**PVY1_IP**	53842264	7.11	98.87	95.40	41.58	90.00
**PVY2**	54788844	6.28	99.05	95.98	44.56	90.92
**PVY2_IP**	53672090	7.11	98.78	95.14	41.42	89.28

* Sample: Sample Name; Clean Reads: The number of clean reads after quality control; Clean Bases(G): The data volume after quality control; Clean Q20(%): The proportion of bases with quality greater than Q20 in clean reads; Clean Q30(%): The proportion of bases with quality greater than Q30 in clean reads; Clean GC(%): The average GC content of bases in clean reads; Effective Rate(%): The proportion of clean reads in the original downstream reads.

Table S5. Table of alignment information between quality-controlled sequencing reads and reference genome

Sample	Total reads	Total mapped(%)	Non unique(%)	Unique(%)	Unmapped reads(%)
**Mock_1**	55893764	55647387(99.56)	7712546(13.86)	47934841(86.14)	246377(0.44)
**Mock_1IP**	54776970	54184946(98.92)	5938896(10.96)	48246050(89.04)	592024(1.08)
**Mock_2**	58272040	57985801(99.51)	7535943(13.0)	50449858(87.0)	286239(0.49)
**Mock_2IP**	53958376	53544076(99.23)	6084368(11.36)	47459708(88.64)	414300(0.77)
**PVY1**	54791514	52993837(96.72)	6061268(11.44)	46932569(88.56)	1797677(3.28)
**PVY1_IP**	53826964	52208220(96.99)	4417674(8.46)	47790546(91.54)	1618744(3.01)
**PVY2**	54783974	52978737(96.7)	7049388(13.31)	45929349(86.69)	1805237(3.3)
**PVY2_IP**	53657094	52118008(97.13)	4223374(8.1)	47894634(91.9)	1539086(2.87)

* Sample: Sample Name; Total reads: Total number of clean reads with sequences; Total mapped(%): Total number of sequences that have been aligned to the reference genome and the proportion; Non unique(%): Number of sequences that have more than one alignment position on the reference sequence; unique(%): Number of sequences that have a unique alignment position on the reference sequence; Unmapped reads(%): Number of sequences that could not be aligned to the reference sequence.

Table S6. Quality control statistics for alignment results table

Name	Type	Mapped	Uniq	Filt	Valid
**Mock_1**	Input	55647387	47934841	0	47934841
**Mock_2**	Input	57985801	50449858	0	50449858
**PVY1**	Input	52993837	46932569	0	46932569
**PVY2**	Input	52978737	45929349	0	45929349
**Mock_1IP**	IP	54184946	48246050	0	48246050
**Mock_2IP**	IP	53544076	47459708	0	47459708
**PVY1_IP**	IP	52208220	47790546	0	47790546
**PVY2_IP**	IP	52118008	47894634	0	47894634

* Name: Sample Name; Type: Sample Type; Mapped: Total number of sequences mapped to the reference genome; Uniq: Number of unique sequences identified; Filt: Number of Input reads randomly selected; Valid: Effective data volume after quality control.

Table S7. The distribution data of reads among various gene elements

Sample	TSS_10kb	TSS_5kb	TSS_1kb	5'UTR	CDS	3'UTR	TES_1kb	TES_5kb	TES_10kb	Intergenic	Intron
**Mock_1**	124764	194063	292959	1985479	51531391	5074208	748451	259580	167423	1744874	3883503
**Mock_1IP**	136035	181413	200682	1290008	33789383	15093249	1365485	390205	192111	2234450	4719786
**Mock_2**	134732	206341	311821	2102084	54888750	5431626	807520	277677	178308	1856984	4202267
**Mock_2IP**	133549	181836	201118	1284206	32705598	14933875	1329383	388386	185339	2189975	4657657
**PVY1**	142512	232958	351760	2270395	49392597	5265434	768674	267843	153328	2016643	4157523
**PVY1_IP**	151531	210128	214325	1333138	32195492	16148749	1416963	425189	194596	2446701	5020436
**PVY2**	128432	220292	322405	2154376	47673146	4819824	678585	245078	144410	1854574	3701940
**PVY2_IP**	150557	201846	189368	1184087	30323621	17395461	1484424	440614	201531	2468538	5111396

* Sample: Sample Name; TSS_10kb, TSS_5kb, TSS_1kb: Upstream 1-10kb region of transcription start site; 5'UTR: 5' non-translated region; CDS: Coding region; 3'UTR: 3' non-translated region; TES_1kb, TES_5kb, TES_10kb: Downstream 1-10kb region of transcription termination site; Intergenic: Intergenic region; Intron: Intron region.
